# Mapping the Efficacy and Mode of Action of Ethylzingerone [4-(3-Ethoxy-4-Hydroxyphenyl) Butan-2-One] as an Active Agent against *Burkholderia* Bacteria

**DOI:** 10.1128/AEM.01808-20

**Published:** 2020-09-17

**Authors:** Laura Rushton, Ahmad Khodr, Florence Menard-Szczebara, Jean-Yves Maillard, Sylvie Cupferman, Eshwar Mahenthiralingam

**Affiliations:** a Cardiff School of Biosciences, Cardiff University, Cardiff, United Kingdom; b International Microbiology Department, L’Oréal Research and Innovation, Chevilly-Larue, France; c Cardiff School of Pharmacy and Pharmaceutical Sciences, Cardiff University, Cardiff, United Kingdom; University of Manchester

**Keywords:** *Burkholderia*, ethylzingerone, genomics, mechanisms of resistance, preservative

## Abstract

Burkholderia cepacia complex (Bcc) bacteria are intrinsically antimicrobial-resistant opportunistic pathogens and key risk species in the contamination of nonfood industrial products. New agents and formulations to prevent growth of *Burkholderia* in home care (cleaning agents) and personal-care (cosmetics and toiletries) products are required. We characterized how ethylzingerone [4-(3-ethoxy-4-hydroxyphenyl) butan-2-one] (HEPB) acts as a preservative with activity against *Burkholderia* species encountered in industry. *Burkholderia* (*n* = 58) and non-*Burkholderia* (*n* = 7) bacteria were screened for susceptibility to HEPB, and its mode of action and resistance were determined for a model Burkholderia vietnamiensis strain using transposon mutagenesis, transcriptomics, and genome resequencing analysis. The susceptibility of *Burkholderia* spp. to HEPB (MIC = 0.45% ± 0.11% [wt/vol]; MBC = 0.90% ± 0.3% [wt/vol]) was characterized, with limited inter- and intraspecies differences. HEPB (1% [wt/vol]) was rapidly bactericidal, producing a 6-log reduction in viability within 4 h. Spontaneous resistance to HEPB did not develop, but transient phenotypes with altered growth characteristics and susceptibility to antibiotics were identified after prolonged exposure to sublethal HEPB concentrations. Transposon mutagenesis and RNA-sequencing analysis identified multiple genetic pathways associated with HEPB exposure, including stress response mechanisms, altered permeability, regulation of intracellular pH, damage and repair of intracellular components, and alteration and repair of lipopolysaccharides. Key pathways included the stringent response, homeostasis of intracellular pH by the *kdp* operon, protection against electrophiles by KefC, and repair of oxidized proteins by methionine sulfoxide reductase enzymes. In summary, we show that HEPB has potent, targeted efficacy against *Burkholderia* bacteria without promoting wider stable antimicrobial resistance. The mode of action of HEPB against *Burkholderia* is multifactorial, but killing by intracellular oxidation is a key mechanism of this promising agent.

**IMPORTANCE** *Burkholderia* bacteria are opportunistic pathogens that can overcome preservatives used in the manufacture of nonsterile industrial products and occasionally cause contamination. Consequently, new preservatives to prevent the growth of key risk Burkholderia cepacia complex bacteria in nonfood industrial products are urgently required. Here, we show that ethylzingerone is active against these problematic bacteria, killing them via a multifactorial mode of action which involves intracellular oxidation.

## Abstract (precis)

*Burkholderia* bacteria are opportunistic pathogens that can overcome preservatives used in the manufacture of nonsterile industrial products and occasionally cause contamination. Consequently, new preservatives to prevent the growth of key risk Burkholderia cepacia complex bacteria in nonfood industrial products are urgently required. Here, we show that ethylzingerone is active against these problematic bacteria, killing them via a multifactorial mode of action which involves intracellular oxidation.

## INTRODUCTION

Antimicrobials are used extensively in health care, domiciliary, agricultural, and industrial settings to inhibit proliferation of spoilage organisms and kill potential pathogens. In industry, combinations of antimicrobials are incorporated at low levels into raw materials and finished products as preservative agents to protect against microbial contamination. Failure of these preservative systems can result in significant economic loss and, depending on the contaminant, may pose a risk to consumer health. A diverse range of yeasts, molds, and bacteria are encountered as contaminants of industrial products. Gram-negative bacteria are commonly encountered in the pharmaceutical, cosmetics, and personal care industries, with a predominance of *Pseudomonas* spp. and Burkholderia cepacia reported in product recalls (1). As it is difficult to differentiate species by conventional phenotypic or biochemical tests, *Burkholderia* contaminants are routinely recorded as “B. cepacia” in these incident reports. This obscures the diversity of *Burkholderia* species actually encountered in the industrial environment. Multiple *Burkholderia* species have been isolated from nonfood industrial products, with a predominance of species from the Burkholderia cepacia complex (Bcc) (2). The Bcc comprises over 20 closely related, but genetically distinct, species within the diverse genus *Burkholderia*. As highly adaptable environmental bacteria, members of the Bcc have been studied for their potential biotechnological applications in plant promotion, bioremediation, and biological control of plant pests (3). In parallel to their beneficial properties, however, members of the Bcc have been extensively studied as opportunistic pathogens capable of causing infection in multiple hosts, such as chronic respiratory infection in people with cystic fibrosis (CF) (4). As industrial contaminants, Bcc bacteria have been isolated from petroleum products (5), antimicrobial solutions (6), pharmaceuticals, and preserved cosmetics and toiletries (1, 7). Outbreaks of Bcc infection in vulnerable individuals, although rare, have resulted from the use of contaminated industrial products (6, 8), and these bacteria have gained recognition as key risk species in microbial contamination (1, 9).

The ability of Bcc bacteria to survive as contaminants is in part due to high innate antimicrobial resistance, and their metabolic flexibility and adaptability (3). A recent survey characterizing the susceptibility of the Bcc to key groups of preservatives used in industry revealed inter- and intraspecies differences in susceptibility and highlighted the observation that the permitted levels of sodium benzoate and benzethonium chloride were ineffective in the control of Bcc contamination (2). The study also demonstrated that *Burkholderia* bacteria can develop stable adaptations to biocides via repeated exposure to sublethal concentrations of preservatives as priming agents (2). Adaptation to key preservatives resulted in derivatives with decreased preservative susceptibility and altered antibiotic susceptibility profiles that persisted in the absence of the priming biocide (2). Transcriptomic analysis of Burkholderia lata, a commonly encountered industrial contaminant, revealed that efflux by a resistance-nodulation-division (RND) system played a key role in adaptation to isothiazolinone agents. In addition, Bcc strains isolated from industrial sources demonstrated increased tolerance to a formaldehyde-releasing agent. Therefore, the selection and emergence of antimicrobial tolerant Bcc bacteria in industry are of concern (2).

There is an unmet need for preservatives that, when used at low levels, are efficacious against intrinsically resistant *Burkholderia* bacteria. Ethylzingerone, which is also referred to as hydroxyethoxyphenyl butanone (HEPB), is a novel cosmetic ingredient recently regulated by the European Union (EU) as a preservative in rinse-off, oral-care, and leave-on cosmetic products at levels of ≤0.7% (wt/vol) (10–12). This phenolic derivative is structurally similar to zingerone [4-(4-hydroxy-3-methoxyphenyl) butan-2-one], which is derived from bioactive molecules (such as gingerols) found in the root of the ginger plant (*Zingiber officinale*). Zingerone is well characterized for its anti-inflammatory activity (13). In a recent investigation, zingerone was shown to have activity against Gram-negative *Pseudomonas* spp. (14). Our working hypothesis, that HEPB also had antimicrobial activity against bacteria of this phylum, including *Burkholderia* species, warranted further investigation. We carried out an exploration of HEPB susceptibility, utilizing a diverse panel of Bcc bacteria, other *Burkholderia* species, and reference non-*Burkholderia* bacteria. Burkholderia vietnamiensis strain G4 (LMG 22486), a key species seen in industrial contamination (2), was used as a model strain to study adaptation to HEPB and the genetic basis for tolerance to the preservative. We defined stable adaptation as a nontransient change in phenotype, specifically antimicrobial susceptibility, that persisted in the absence of the priming preservative agent. Transposon mutagenesis and transcriptomic analysis of *B. vietnamiensis* strain G4 identified key genes and pathways involved in HEPB susceptibility and revealed its multifactorial mode of action against *Burkholderia* bacteria.

## RESULTS

### *Burkholderia* susceptibility to HEPB.

The MICs and minimum bactericidal concentrations (MBCs) of HEPB for 58 *Burkholderia* strains (Table S1), representative of species commonly encountered as industrial contaminants, and 7 reference non-*Burkholderia* isolates (Table S2) were evaluated. Data for individual strains are available in Tables S3 and S4. The 58 *Burkholderia* strains belonged to 22 species, with 54 strains belonging to 20 species of the Bcc. In this experimental system, HEPB solubility prevented the evaluation of test concentrations above 2% (wt/vol). Nearly a quarter of the *Burkholderia* strains (24%; *n* = 14) were killed by 0.7% (wt/vol) HEPB (Table S3), which is the maximum concentration permitted for use in rinse-off, oral-care, and leave-on cosmetic products by EU Regulation 1223/2009, Annex V (10). The mean MICs and MBCs of HEPB demonstrated little variation for *Burkholderia* strains (Table 1). The majority of the strains had a median MIC of 0.5% (wt/vol) (48 strains; 83%), and 44 strains (76%) had a median MBC of 1% (wt/vol) (Table S3). The largest variation in susceptibility within a species group was a 4-fold difference in MICs for three Burkholderia ambifaria strains (ranging from 0.125% to 0.5% [wt/vol]) (Table S3).

**TABLE 1:** MIC and MBC of HEPB for *Burkholderia* and *Paraburkholderia* strains^*a*^

Genus or group (*n*)	HEPB MIC (% [wt/vol])		HEPB MBC (% [wt/vol])	
	Median	Mean ± SD	Median	Mean ± SD
*Burkholderia* (58)	0.5	0.45 ± 0.12	1	0.90 ± 0.30
Bcc (54)	0.5	0.46 ± 0.11	1	0.91 ± 0.29
*Paraburkholderia* (2)	0.125	0.125	0.5	0.625 ± 0.25

a Bcc, Burkholderia cepacia complex; *n*, number of strains. Median and mean values were derived from three biological replicate experiments. MIC and MBC data for individual strains are available in Table S3.

The MICs and MBCs of the non-*Burkholderia* panel were more variable (see Table S4). Pseudomonas aeruginosa, Staphylococcus aureus, and Escherichia coli strains were not killed by 2% (wt/vol) HEPB, the limit of the agent’s solubility, and therefore, MICs and MBCs could not be determined. *Paraburkholderia* strains (Table S2) had increased susceptibility to HEPB compared with *Burkholderia* strains, as shown in Table 1.

The preservative susceptibility of 39 *Burkholderia* strains utilized in this study was published previously by Rushton et al. (2). Metadata analysis shows that in comparison to other established preservatives, the effectiveness of industrially relevant levels of HEPB against *Burkholderia* bacteria is similar to that of methylparaben or phenoxyethanol (Fig. S1). Inhibitory concentrations of HEPB for the 39 *Burkholderia* strains ranged from 1.4- to 5.6-fold lower than the EU maximum permitted level of 0.7% (wt/vol), while inhibitory concentrations of methylparaben and phenoxyethanol were 4 to 8-fold lower than maximum regulated levels of 0.4% (wt/vol) and 1% (vol/vol), respectively.

**FIG 1:**
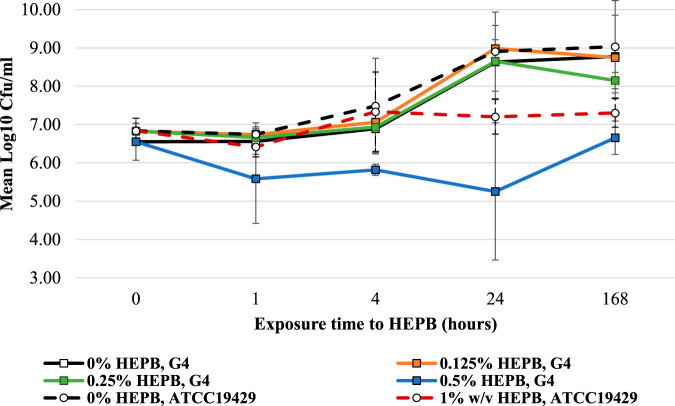
Time-kill curves of *B. vietnamiensis* strain G4 and P. aeruginosa ATCC 19429 cultured in TSB with HEPB at concentrations below in-use levels. *B. vietnamiensis* strain G4 viability decreased to undetectable levels after exposure to 1% (wt/vol) HEPB for 1 h (data not shown). P. aeruginosa ATCC 19429 is indicated by dashed lines. Cultures were sampled over 1 week and neutralized before enumeration of viable cells. Data are means ± standard deviations (SD) for three biological replicates. The lower detection threshold was 10^3^ CFU/ml. A final concentration of 2% (vol/vol) DMSO control did not reduce cell viability.

*B. vietnamiensis* strain G4 was an ideal model strain to evaluate HEPB mode of action and resistance, as it possessed an intermediate susceptibility to HEPB, with an MIC of 0.25% (wt/vol) and MBC of 0.5% (wt/vol). Time-kill curves of *B. vietnamiensis* strain G4 exposed to HEPB demonstrated the rapid bactericidal activity of HEPB, with a 6-log reduction in viability occurring within an hour of exposure to 1% (wt/vol) HEPB. In contrast, P. aeruginosa ATCC 19249 did not show a reduction in viability at this concentration of HEPB (Fig. 1). Confocal microscopy of *B. vietnamiensis* strain G4 exposed to HEPB concentrations varying from 0.125% to 1% (wt/vol) for 18 h revealed that cell death increased in a dose-dependent manner (Fig. S3). At 1% (wt/vol) HEPB, only fragments of nonviable bacterial cells, as determined by total viable count (TVC), were observed by a live/dead stain (Fig. S3).

### Spontaneous resistance and adaptation of *B. vietnamiensis* strain G4 to HEPB.

**FIG 2:**
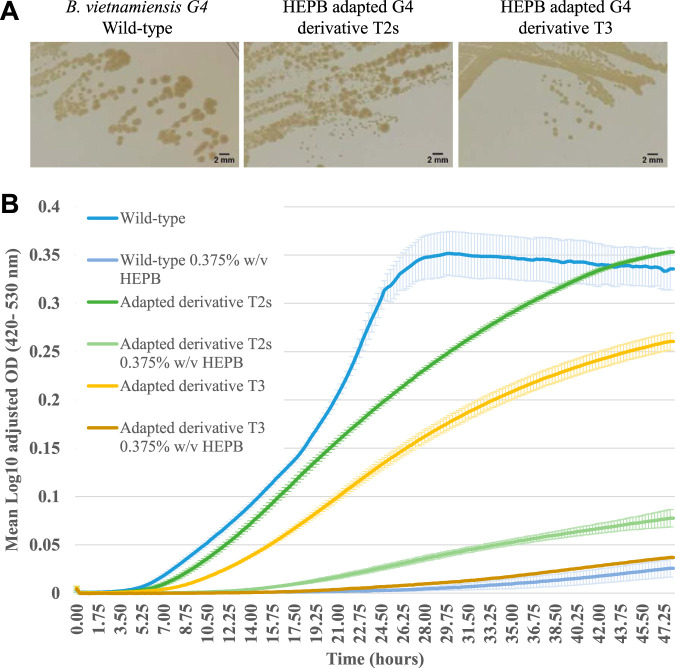
Colony morphology and growth curve of *B. vietnamiensis* wild-type strain G4 and HEPB-adapted derivatives T2s and T3. (A) Colony morphology of wild-type (parental) strain and HEPB-adapted derivates cultured on TSA without HEPB for 24 h. HEPB-adapted derivatives form smaller discrete round colonies than the wild type. Bar, 2 mm. (B) Growth of the wild type and HEPB-adapted derivatives in TSB without HEPB and 0.375% (wt/vol) HEPB. Data are means ± SD for two biological replicates. Growth of T2s is similar to that of the wild-type in the absence of HEPB. HEPB-adapted derivatives reached higher final ODs than the wild-type in the presence of HEPB. Data for HEPB-adapted derivative T2L are not shown, as its colony morphology and growth in the absence and presence of HEPB were similar to those of the wild type.

The propensity of *Burkholderia* to develop resistance to HEPB was investigated using *B. vietnamiensis* strain G4. The frequency of spontaneous resistance to HEPB was calculated as zero, as none of the ∼1 × 10^6^ CFU plated onto agar containing 2× MIC (0.5% [wt/vol]) of HEPB grew as resistant colonies. Adaptation to HEPB was developed via the progressive subculture of the parental strain G4 on agar with subinhibitory HEPB concentrations. Stable adaptation was defined as a change in HEPB susceptibility and phenotype that persisted in the absence of HEPB. The resulting three HEPB-adapted derivatives of *B. vietnamiensis* G4 (named T2s, T2L, and T3) did not demonstrate a large decrease in susceptibility to HEPB: all three had an MIC of 0.375% (wt/vol) (1.5-fold higher than the parental G4 strain). Derivatives T2s and T3 also had altered colony morphology with smaller discrete colonies than that of the parental strain (Fig. 2A). Such changes in morphology and susceptibility were not observed after the equivalent serial passage of the parental strain in culture medium without HEPB. In the absence of HEPB, adapted derivatives demonstrated a lower rate of growth than that of the parental strain. The HEPB-adapted derivative T3 achieved a significantly lower final optical density than the parental strain when cultured for 48 h in the absence of HEPB (mean log_10_ optical density [OD], 0.260 ± 0.011 and 0.336 ± 0.023, respectively; *P* < 0.05) (Fig. 2B). In culture with 0.375% (wt/vol) HEPB, adapted derivative T2s achieved a significantly higher final OD than that of the parental strain (mean log_10_ ODs, 0.078 ± 0.01 and 0.026 ± 0.011, respectively; *P* = 0.05), whereas its final OD in the absence of HEPB was similar to that of the parent (Fig. 2B). HEPB-adapted derivatives had varied degrees of altered susceptibility to eight antibiotics that were representative of agents active against different cellular targets (Table 2). To evaluate the stability of HEPB adaptation and antibiotic susceptibility, derivatives were subcultured repeatedly in the absence of HEPB. After serial passage, susceptibility to ceftazidime, imipenem, piperacillin, and ciprofloxacin reverted to wild-type levels; sensitivities to amikacin and azithromycin were also closer to that of the parental strain. Increased tolerance to chloramphenicol in HEPB-adapted derivatives persisted after serial passage in the absence of HEPB).

**TABLE 2:** Antibiotic susceptibility profiles of HEPB-adapted *B. vietnamiensis* strain G4 derivatives^*a*^

Strain	Mean MIC (μg/ml) of:							
	AMK	AZM	CIP	CHL	IPM	PIP	SXT	CAZ
Parent (G4)	1	4	0.19	12	0.125	0.5	0.125	0.5
Derivative T2s	1	4	0.19	32	0.125	0.5	0.19	0.38
Derivative T2L	1	3	0.19	12	0.125	0.5	0.19	0.5
Derivative T3	0.25	1	0.64	24	0.16	0.25	0.125	0.25

a Abbreviations: AMK, amikacin; AZM, azithromycin; CIP, ciprofloxacin; CHL, chloramphenicol; IPM, imipenem; PIP, piperacillin; SXT, trimethoprim-sulfamethoxazole; CAZ, ceftazidime. The antibiotic susceptibility profiles of HEPB-adapted derivatives before serial passage in the absence of HEPB are shown.

Whole-genome resequencing and comparison to the parental *B. vietnamiensis* G4 genome (8.4 Mb in size) revealed limited (*n* = 9) nucleotide polymorphisms in coding sequences (CDS) across the genomes of the HEPB-adapted derivatives. Three missense variants with putative function were common to all three derivatives: His102Tyr in a putative voltage-gated CIC-type chloride channel (gene Bcep1808_1681); Gln1527Arg in a putative modification methylase (gene Bcep1808_7553); and Lys21Glu in a putative purine nucleoside phosphoramidase (gene Bcep1808_0414). Derivatives T2s and T3 shared the missense variant Arg222Cys in a putative murein hydrolase activator (gene Bcep1808_1748). Derivative T3 had the missense variant Cys266Gly in a DNA-dependent RNA polymerase (gene Bcep1808_0356). The remaining nucleotide polymorphisms were synonymous variants that did not change the encoded amino acid.

### Mapping *B. vietnamiensis* strain G4 genes associated with HEPB susceptibility by transposon mutagenesis.

A single mutant bank of 3,984 derivatives of *B. vietnamiensis* strain G4 was created using the transposon mini-Tn*5-luxCDABE*-Km (15). The initial phenotypic screen by agar dilution assay identified 1,229 mutants (30.8% of the bank) with increased or decreased HEPB susceptibility. The 1,229 mutants were rescreened by broth dilution assay, and a subset of 46 mutants with increased or decreased HEPB susceptibility were selected for detailed growth analysis in the presence and absence of the preservative. Growth curve analysis revealed that 18 of the 46 mutants reached a lower final mean OD (log_10_-adjusted OD, ≤0.25) than that of the wild-type strain G4 (log_10_-adjusted OD, ≥0.35) under control conditions. Consequently, these 18 mutants were excluded from further characterization.

The genetic context was determined for 28 mutants that displayed a change in HEPB susceptibility but maintained wild-type levels of growth under control conditions (Table 3). Transposon insertion had occurred at random, covering the three main genomic replicons and five plasmids of strain G4. *B. vietnamiensis* strain G4 genes associated with HEPB susceptibility were involved in a range of putative functions; the predominant COG (Clusters of Orthologous Groups) categories were transcription (*n* = 5), unknown function (*n* = 4), and signal transduction (*n* = 3) (Table 3). Mutation of genes involved in the regulation of intracellular pH, the response to external chemical stimuli or adverse conditions, and the response to oxidative stress increased HEPB susceptibility. A 4-fold decrease in HEPB MIC (0.0625% (wt/vol) by agar dilution assay) was associated with mutation of a *spoT* homolog (Bcep1808_0918, mutant 22:E11). This gene putatively encodes a bifunctional (p)ppGpp synthase/hydrolase, a mediator of the stringent response, which coordinates a variety of cellular activities in response to change or adverse conditions (16). Also associated with the response to extracellular stimuli, the mutation of gene Bcep1808_3929 (mutant 35:G5) resulted in increased HEPB susceptibility. This putative sensor hybrid histidine kinase has a predicted protein-protein interaction with an osmolality response regulator homologous to *ompR* (17).

**TABLE 3:** Transposon-interrupted genes of *B. vietnamiensis* strain G4 exhibiting altered susceptibility to HEPB and wild-type growth under control conditions

Mutant ID	G4 DNA flanking transposon insertion site (20 bp)	Gene ID	Replicon	Mutated gene and putative function^*a*^	COG category^*b*^
9:G3	ACCCATCACCATGCCCACA	4698	chr2	*ilvD*; dihydroxy-acid dehydratase	Amino acid transport and metabolism
44:B9	CGTTCCGGCGCGGCGCTGCC	6500	chr3	Cellulose synthase domain-containing protein	Carbohydrate transport and metabolism
42:E3	AAGTAAGACAGGTCACGAAC	1471	chr1	RND efflux system outer membrane lipoprotein	Cell wall/membrane/envelope biogenesis
14:C2	AGCAGTTCATCGCGCTGGC	0037	chr1	Type III restriction enzyme, res subunit	Defense mechanisms
9:D8	ATCGCTGACCACCCGCGCT	0174	chr1	Hypothetical protein (putative restriction endonuclease)	Defense mechanisms
5:D5	CGGCTAGGCGGCCAGATCT	3088	chr1	*puuB*; gamma-glutamylputrescine oxidoreductase	Energy production and conversion
43:B11	AGGAGAAAGGCCCCGTCATC	3033	chr1	2-Oxoacid ferredoxin oxidoreductase	Energy production and conversion
6:F3	AGGCGGCCAGATCTGATCA	7566	pBVIE04	Hypothetical protein	Function unknown
9:D7	CCCCCCCGTACTAGTCGAC	3807	chr2	Amine oxidase	Function unknown
19:E4	CGCTGGCGGCCAGATCTGA	1415	chr1	Hypothetical protein	Function unknown
44:E6	GTCAACGCGTGGCCAAATCG	6803	pBVI01	Hypothetical protein	Function unknown
29:B7^*c*^	CGGCTAGGCGGCCAGATCT	2890	chr1	*kefC*; glutathione-regulated potassium-efflux system protein	Inorganic ion transport and metabolism
27:G3	CCCCCGCCGTACTAGTCGA	5515	chr3	*msrA*; peptide methionine sulfoxide reductase	Posttranslational modification, protein turnover, chaperones
19:D4	CTGCTCCGGCACGACGTCCA	0426	chr1	DNA primase TraC	Replication, recombination and repair
42:B11	ATAATAGTCAAGGCGTGGCC	6093	chr3	Transposase Tn*3* family protein	Replication, recombination and repair
7:G7	TGAGTTTAATGTCTTCGCT	6805	pBVIE01	Putative signal transduction protein	Signal transduction mechanisms
19:H7	CCGACGCGCGCCGGCAGCG	2370	chr1	*kdpD*; sensor protein	Signal transduction mechanisms
35:G5	CGTGACCAGGTGCTCGCGA	3929	chr2	Integral membrane sensor hybrid histidine kinase	Signal transduction mechanisms
8:D10	GGCAGGCCAGATCTGATCA	7553	pBVIE04	Helicase domain-containing protein	Transcription
6:F4	CGTGACGACCGAGTCGAAG	3650	chr2	Chromosome replication initiation inhibitor protein	Transcription
43:G1	CGCTCACTGCCGGCCGGCAA	4632	chr2	LysR-family transcriptional regulator	Transcription
20:G12^*c*^	TACGACCAGTCTGCGAATCG	3794	chr2	*noc*; nucleoid occlusion (*parB*-like) protein	Transcription
22:E11^*c*^	TCAGCTAGGCGGCCAGATCT	0918	chr1	*spoT*; bifunctional (p)ppGpp synthase/hydrolase	Transcription, signal transduction mechanisms

a Putative function of poorly characterized proteins based on predicted protein-protein interactions networks in the STRING database (17).

b Identified using the eggNOG database (65).

c Mutant demonstrated a 4-fold decrease in MIC (0.0625% [wt/vol]) of HEPB by agar dilution assay. All other mutants demonstrated a 2-fold decrease in MIC of HEPB by agar dilution assay.

Mutation of two genes involved in the regulation of intracellular pH increased HEPB susceptibility: gene Bcep1808_2370 (mutant 19:H7), homologous to *kdpD*, a member of a two-component regulatory system of the *kdp* operon, which encodes a high-affinity potassium (K^+^) transporter; and a homolog of *kefC* (Bcep1808_2890; mutant 29:B7), a potassium/sodium antiporter efflux system that confers protection against externally derived electrophiles (18). Mutation of a third gene, with putative involvement in intracellular homeostasis and polyamine-mediated protection against oxidative stress, also resulted in increased HEPB susceptibility. Gene Bcep1808_3088 (mutant 5:D5), homologous to *puuB*, is a putative gene for gamma-glutamylputrescine oxidoreductase involved in putrescine catabolism, a precursor of the polyamines spermidine and spermine (19). A large increase in HEPB susceptibility (confirmed by growth curve analysis) was associated with the mutation of a putative *msrA1* gene homolog (Bcep1808_5515; mutant 27:G3). Methionine sulfoxide reductase (Msr) enzymes are involved in the repair of proteins inactivated by oxidation (20). Interruption of key genes associated with transcription, and DNA replication or repair, also changed HEPB susceptibility (Table 3). A large increase in HEPB susceptibility (4-fold reduction in MIC) resulted via the mutation of gene Bcep1808_3794 (mutant 20:G12). This homolog of *noc* that putatively encodes a *parB*-family protein has involvement in nucleoid occlusion and is recognized as a transcriptional regulator of stress response genes in P. aeruginosa (21).

### Mapping *B. vietnamiensis* global gene expression in the presence of HEPB.

RNA-sequencing analysis was used to identify differential gene expression of *B. vietnamiensis* strain G4 in response to subinhibitory concentrations of HEPB. Growth curve analysis revealed that strain G4 was able to grow in the presence of 0.2×, 0.5×, and 0.75× MIC of HEPB, but with significantly (*P* < 0.05) altered growth kinetics in comparison to control conditions (Fig. S2). This meant that final optical density (OD) values of the considered test concentrations were decreased by 22%, 55%, and 68%, respectively (MIC reduced mean final OD by 82%) in comparison to that of the control at 24 h (Fig. S2). A test concentration of 0.5× MIC (0.125% [wt/vol] HEPB), sampled at 8 h, was utilized for gene expression analysis, as the cultures had significantly altered growth but consistently reached a density that yielded high-quality RNA from live cells (determined by viable count) and a growth rate equivalent to that of the strain cultured in parallel under control conditions.

Paired-end sequence reads (ranging from 1.82E + 06 to 3.16E + 06 in total, for the control and test conditions) were aligned to the reference *B. vietnamiensis* strain G4 genome. A mean of 94.35% (range, 96% to 93%) of the sequence reads were found to map to coding sequences. Differential gene expression occurred in response to HEPB at subinhibitory concentrations: 21.94% of the *B. vietnamiensis* strain G4 genome had significantly altered expression, including 189 upregulated genes (Table S5) and 70 downregulated genes (Table S6) with a significant log_2_ fold change of ≥1.5. In total, 18 operons were significantly upregulated and eight were downregulated, with a log_2_ fold change of ≥1.5 (*P* < 0.05). This included the upregulation of two RND efflux systems, with up to +1.89-fold changes in expression (Table S5), and three operons involving transposable elements with homology to IS30-, TniB-, and Tn*3*-family proteins (present on the plasmid and the chromosome), with up to +3.05-fold changes in expression. Additionally, many integrases distributed along the genome of strain G4 were upregulated (Table S5). Expression of two copies of H-NS, a global regulator and a xenogeneic silencer (22–24), was also upregulated.

The largest significant change in expression (+4.26-fold) was associated with the gene Bcep1808_2705, which putatively encodes a sorbitol dehydrogenase. Network analysis of KEGG pathways indicated that the operon was putatively involved in fatty acid biosynthesis (17). All three genes within the operon were significantly upregulated ≥+2.85-fold in response to HEPB. In connection, genes encoding a sorbitol-binding extracellular binding protein (Bcep1808_2709) and the operon from Bcep1808_2705 to Bcep1808_2708, which encodes an ABC transporter system, were significantly upregulated, 3.32- to 4.06-fold, in response to HEPB (Table S5). Twenty-five of the genes upregulated by ≥2-fold were located on plasmid pBVIE02. The majority of these genes (with putative function) were involved in transcription, replication, recombination and repair, including a *parB* homolog and several transposable elements.

Genes significantly downregulated ≥1.5-fold in response to HEPB were located on chromosome 1 (*n* = 29), chromosome 2 (*n* = 23), chromosome 3 (*n* = 7), and plasmids pBVIE01 (*n* = 10) and pBVIE03 (*n* = 1). These genes were predominantly of unknown function (*n* = 19) or had a putative role in intracellular trafficking and secretion (*n* = 10), amino acid transport (*n* = 8), or energy production (*n* = 8) (Table S6). This included a −2.64- to −1.67-fold change in an operon of genes encoding a putative type II secretion system (Bcep1808_1487 to Bcep1808_1491, chromosome 1) involved in protein secretion. Genes involved in the high-affinity binding and transport of the aliphatic branched-chain amino acids leucine, isoleucine, and valine were downregulated ≤−2.84-fold (Bcep1808_6683, Bcep1808_6691, and Bcep1808_3370, respectively). Two porins were significantly downregulated by −2.08 and −2.28-fold (genes Bcep1808_4025 and Bcep1808_4974, respectively).

## DISCUSSION

Manufacturers strive to formulate robust preservative systems with a wide antimicrobial spectrum that prevent microbial growth and will not lead to the development of resistant microorganisms. Although there are currently over 100 chemical substances regulated as primary synthetic or natural preservatives for use in personal-care products, toiletries, and cosmetics, under EU Commission Regulation 1223/2009, Annex V (25), this challenge to manufacturers is compounded by tighter regulations on preservative limits of use and by consumer pressure for milder preservation in personal-care and cosmetic products. As a result, the most commonly used preservative agents fall into just 11 groups by chemical composition (26). In recent years, safety concerns over estrogenic activity and sensitization have led to a significant reduction in formulations containing parabens and isothiazolinone preservatives, both highly efficacious agents against Bcc bacteria (2). Currently, the industry suffers from a considerable lack of less-toxic preservatives with a potent antimicrobial activity against these key contaminants.

### HEPB is efficacious against key risk Burkholderia cepacia complex bacteria.

HEPB demonstrated good antimicrobial activity against a diverse panel of 58 *Burkholderia* strains representing species commonly encountered as contaminants. In contrast to previous antibiotic, biocide, and preservative susceptibility surveys (2, 27), there were few or no inter- and intraspecies differences in HEPB susceptibility, with the majority of strains being inhibited by the industrially relevant level of 0.5% (wt/vol). Greater inter- and intraspecies differences in HEPB susceptibility may well become apparent in a larger strain collection. However, this finding suggests that the antimicrobial activity of HEPB exploits a vulnerability shared by *Burkholderia* species. The majority of *Burkholderia* strains evaluated were inhibited, and 24% were killed, by the maximum permitted level of HEPB for use in EU cosmetics and toiletries. The targeted antimicrobial potency against *Burkholderia* species was in stark contrast to the HEPB susceptibility of the non-*Burkholderiales* bacteria evaluated. In this study and a recent *in vitro* investigation by Wesgate et al. (28), industrially relevant levels of HEPB were subinhibitory for the same P. aeruginosa, E. coli, and S. aureus reference strains. Even at 2% (wt/vol), HEPB was nonlethal for these bacteria. However, it is noteworthy that the HEPB susceptibility of other genera has not yet been systematically surveyed with taxonomically diverse test strain panels. This study also investigated the inherent activity of HEPB in an aqueous solution with the cosolvent dimethyl sulfoxide (DMSO). In application, HEPB will be deployed in product formulations containing surfactants, sequestrants, and other compounds that can interact with cellular targets and may affect, or even potentiate, its antimicrobial potency (29).

### *B. vietnamiensis* adaptation to HEPB is transient and does not confer high-level HEPB resistance.

Concerns regarding the potential link between widespread biocide use and the emergence of antimicrobial-resistant microorganisms have been voiced for many years. In support of recent opinions from scientific committees (30), the EU now requires that manufacturers of biocidal products provide information on the development of resistance to their products in target organisms (31). The challenge to manufacturers of biocides and preserved nonfood products is to predict resistance development in formulations using low-cost high-throughput techniques that reflect in-use conditions (32). Bcc bacteria have been shown to increase their antibiotic resistance after selection for spontaneous resistance (33) and to increase tolerance to isothiazolinone and benzethonium chloride preservatives via adaptive resistance (2). In both studies, stable gene expression changes and cross-resistance to other antimicrobials were shown to persist in the absence of selective pressure or priming agents. However, changes in antibiotic susceptibility were not clinically relevant. The propensity for Bcc bacteria to develop resistance to HEPB was therefore carefully considered, as both spontaneous resistance and adaptation could contribute to the emergence of resistant organisms.

Spontaneous resistance, which occurs naturally in culture, did not develop to HEPB, even at a concentration below the in-use recommendation (0.5% [wt/vol]). This may have been the result of a multifactorial mode of action and/or the absence of mutation(s) accumulating in a specific cellular target or resistance mechanism. Unlike the previously observed stable adaptation of *Burkholderia* spp. to isothiazolinone and benzethonium chloride preservatives (2), adaptation to sublethal concentrations of HEPB was transient. The susceptibility of HEPB-adapted *B. vietnamiensis* G4 derivatives to HEPB and seven of the eight antibiotics evaluated reverted to wild-type levels in the absence of the priming agent. The mechanism(s) responsible for the elevated tolerance to chloramphenicol in HEPB-adapted derivatives, which persisted even in the absence of HEPB, was not elucidated. However, the contribution of efflux pump activity to chloramphenicol resistance in Burkholderia cepacia complex species is well documented (34). Wesgate et al. (28) also reported HEPB to have a low propensity to induce phenotypic resistance in other genera; short-term exposure to HEPB (24 h, in a tryptone and sodium chloride suspension) did not change the antimicrobial susceptibility profile of P. aeruginosa or S. aureus strains, although E. coli became susceptible to gentamicin. The propensity of formulated HEPB to induce resistance in these genera was not investigated. Studies suggest that the frequency and extent of decreases in susceptibility to preservatives can be significantly lower when the biocides are incorporated into product formulation (29, 32, 35).

Genomic analysis of the HEPB-adapted derivatives revealed that few DNA polymorphisms resulted from the prolonged exposure of *B. vietnamiensis* to subinhibitory levels of HEPB. This finding supports the opinion of the Scientific Committee on Consumer Safety (SCCS) that, based on bacterial reversion mutation tests in *Salmonella* spp., HEPB is not a potential mutagen (10). The low rate of mutation in HEPB-adapted *B. vietnamiensis* derivatives suggests that the transient changes in phenotype and antimicrobial susceptibility were a result of gene expression changes and/or the epigenetic regulation of gene expression at a transcriptomic level (36). Reversible changes to antimicrobial susceptibility have been attributed to transient phenotypic adaptations, such as the induction of bacterial stress responses (37) or nonspecific reductions in the cellular permeability (38), that revert to pre-exposure levels once the antimicrobial is removed (29). DNA methylation has been shown to epigenetically regulate gene expression in Burkholderia cenocepacia (39), playing an important role in biofilm formation and motility. The role of DNA methylation in the preservative resistance of *Burkholderia* spp. remains to be determined. The “methylome” of *B. vietnamiensis* strain G4 was not characterized in this study, but results suggest that key genetic pathways associated with HEPB may be regulated by DNA methylation. The mutation of gene Bcep1808_0037 (mutant 14:C2) (Table 3), which encodes a highly conserved type III methyltransferase orthologous to B. cenocepacia J2315 gene BCAL3494 (39), resulted in a 2-fold decrease in MIC for HEPB.

### The HEPB mode of action against Bcc bacteria is multifactorial.

A large global gene expression effort was required for *B. vietnamiensis* strain G4 to grow in the presence of HEPB at half the MIC. Key genetic pathways, identified by transposon mutagenesis and transcriptomic analysis, suggested that HEPB had a multifactorial mode of action and did not target a specific cellular target. The highest significantly upregulated gene pathways in response to HEPB were associated with fatty acid biosynthesis pathways (17) involved in fructose and mannose metabolism. Lipids are major targets during oxidative stress (40); therefore, the effect of HEPB on the outer membrane and lipopolysaccharides of *Burkholderia* bacteria warrants further investigation.

Multiple key gene pathways associated with HEPB were involved in stress responses, and survival of adverse conditions. This included *parB* gene homologs, shown to regulate stress response in *Pseudomonas* (21), and *spoT*, a mediator of the stringent response shown to influence expression of numerous genes with an effect on bacterial cell physiology that impacts antimicrobial susceptibility (41). Mutation of *relA* or *spoT* in P. aeruginosa has been shown to increase susceptibility to antimicrobials that cause oxidative stress (16). Transposable elements within *Burkholderia* have also been shown to have increased activity in response to oxidative stress (42). The role of transposable elements in HEPB susceptibility remains to be elucidated. The activity of these elements may also influence the regulation of genes and modulate the organisms’ stress response(s), an epigenetic effect that has been observed in plant cells (43).

Key gene pathways involved in the repair of proteins damaged by oxidation and repair of damaged DNA were associated with HEPB. This suggested that HEPB damages and potentially kills cells via oxidation. Based on its chemistry, the carbonyl group is the likely, albeit weak, electrophilic center of the molecule that would require activation of the oxygen for reactivity. HEPB is not considered an oxidant, and the oxidative damage it causes may result from a reactive metabolite or by an indirect mechanism that produces endogenous reactive oxygen species (ROS) (44). Methionine sulfoxide reductase enzymes (including MsrA) have been shown to be important for resistance against oxidative stress in a range of bacteria (45) and are key participants in maintaining the homeostasis of the cytoplasm and envelope of bacteria. Mutation of the *msrA* gene in *B. vietnamiensis* resulted in increased HEPB susceptibility. This suggests that HEPB causes the oxidation of methionine (Met) residues in *B. vietnamiensis* and that the full repair of these proteins requires the action of both MsrA and MsrB enzymes. Isothiazolinone preservatives, shown to be highly efficacious against Bcc bacteria (2), also target amino acids particularly vulnerable to oxidation. These reactive electrophilic biocides oxidize the thiol (SH) functional group of cysteine residues of cytoplasmic and membrane-bound enzymes and damage DNA at higher concentrations (46).

A key role for the *kdp* operon, encoding a high-affinity K^+^ transporter and KefC potassium channels, suggested that the regulation of intracellular pH was important for the survival of *B. vietnamiensis* exposed to HEPB. KefC efflux systems have been shown to protect E. coli against intracellular damage caused by externally derived electrophiles, via potassium efflux and the rapid acidification of the cytoplasm (47). Other key genes identified suggested that altered cellular permeability and defense mechanisms were potentially required to reduce uptake and intracellular concentrations of HEPB and aid survival. There was a reduction in the expression of porins and increased expression of efflux systems, including those of the RND family. However, these RND systems were not homologs of those previously associated with adaptive resistance to isothiazolinone preservatives (2).

### Conclusions.

In a study of susceptibility and genetic analysis, we have demonstrated that HEPB is active against *Burkholderia* species encountered as industrial contaminants and has a low risk of promoting its own or other antimicrobial resistance in these Gram-negative bacteria. Key genetic pathways associated with HEPB susceptibility were involved in bacterial stress responses and damage repair mechanisms. These indicated that the agent is multifactorial, causing oxidative stress and damage to intracellular components. Overall, this study supports the use of HEPB as an efficacious preservative against *Burkholderia* bacteria, recognized as antimicrobial-resistant and objectionable industrial contaminants.

## MATERIALS AND METHODS

### Bacterial strains and culture conditions.

A panel of 58 *Burkholderia* and 7 non-*Burkholderia* strains used for profiling HEPB susceptibility was drawn from the Cardiff University Collection (3), including reference strains from the Belgium Coordinated Collection of Microorganisms (BCCM) (https://bccm.belspo.be/) (Tables S1 and S2). The collection comprised 20 of the current Burkholderia cepacia complex (Bcc) species groups, reference strains from the Bcc experimental strain panel (48), and 39 strains previously profiled for preservative susceptibility by Rushton et al. (2). Two non-Bcc species, Burkholderia gladioli and Burkholderia plantarii, were also included. The *Burkholderia* panel strains were originally isolated from clinical (*n* = 27), environmental (*n* = 23), and industrial (*n* = 8) sources. Seven non-*Burkholderia* species were evaluated as a control group, including two members of the closely related *Paraburkholderia* clade (4), antibiotic- and biocide-testing reference strains of Staphylococcus aureus and Pseudomonas aeruginosa, and a reference strain of Escherichia coli. For consistent revival, strains were cultured at 30˚C on tryptone soy media (tryptic soy broth [TSB] or tryptic soy agar [TSA]; Oxoid Ltd., United Kingdom). Strains were stored in TSB containing 8% (vol/vol) dimethyl sulfoxide (DMSO) (Sigma-Aldrich, United Kingdom) at −80°C.

### Preservative susceptibility testing.

The MIC and minimum bactericidal concentration (MBC) of HEPB (L’Oréal, France) were determined by standardized agar dilution and broth dilution assays as described by Rushton et al. (2), using TSA and TSB. Bacterial culture was performed at 30°C to be representative of industrial product manufacture and storage. Preservative stock solutions (50% [wt/vol]) were prepared in DMSO (Sigma-Aldrich, United Kingdom), and the required volume was then added to the growth medium to achieve the desired test concentration (range, 0.03125 to 2% [wt/vol] HEPB). Test medium was used on the day of preparation. The final concentration of DMSO in the presence of the bacteria was nontoxic (not exceeding 4% [vol/vol]) and was included as a control condition in assays to rule out its effect on growth. The MIC was defined as the lowest concentration of preservative at which there was an 80% reduction in liquid culture OD_630_, or no visible growth of the test organisms on an agar medium (TSA). The bactericidal activity of HEPB up to 2% (wt/vol) above the maximum concentration for intended use (0.7% [wt/vol]) in rinse-off, oral-care, and leave-on cosmetic products was examined. The MBC was determined as the lowest concentration to elicit a 99% rate of killing, at which growth on recovery medium (TSA) ceased. Preservatives were inactivated prior to the recovery and enumeration of surviving test organisms by dilution in a 1.5% (vol/vol) Tween 80–3% lecithin neutralizing solution as described by Rushton et al. (2). The efficiency and toxicity of the neutralizing solution were evaluated prior to experimentation as described by Lear et al. (49). Three biological replicates, each with three technical replicates, were obtained.

### *Burkholderia* resistance to HEPB.

*B. vietnamiensis* strain G4 (LMG 22486) was used to evaluate the propensity of *Burkholderia* to develop resistance to HEPB. To enumerate spontaneous resistance occurring within a culture, 1 × 10^6^ CFU of strain G4 from a fresh overnight (18-h) TSB culture was inoculated onto the surfaces of replicate TSA plates containing HEPB at concentrations 2-fold higher than the MIC, and mutants with decreased susceptibility were enumerated after 24 h of culture.

A stepwise training assay was performed to select for HEPB-adapted derivatives. Approximately 1 × 10^6^ CFU of an 18-h TSB culture of *B. vietnamiensis* strain G4 was inoculated onto the surface of TSA plates containing HEPB concentrations up to 8-fold lower than that of the MIC as described by Rushton et al.(2). After 24 h culture at 30°C, growth from the starting TSA-HEPB plates was subcultured onto TSA containing a 2-fold increase in HEPB concentration. The serial passage of *B. vietnamiensis* strain G4 on TSA with a gradual increase in HEPB concentrations (to above the MIC) was repeated until growth ceased. HEPB-adapted *B. vietnamiensis* strain G4 derivatives were stored at −80°C as described above.

Stability of the HEPB-adapted phenotype was then evaluated after five passages on TSA plates without HEPB. HEPB-adapted derivatives were confirmed as *B. vietnamiensis* strain G4 by random amplified polymorphic DNA (RAPD) analysis (50).

### Analysis of growth dynamics.

The growth dynamics of the wild-type *B. vietnamiensis* strain G4, HEPB-adapted derivatives of strain G4, and P. aeruginosa (ATCC 19429) were evaluated using a broth dilution assay in a Bioscreen C microbiological growth analyzer (Labsystems, Finland). Starting cultures were standardized by OD as described by Rushton et al. (2), and quadruplicate 200-μl cultures in the multiwell plate were inoculated with 1 × 10^6^ CFU. Turbidity readings were taken using a wide-band filter (450 to 580 nm) every 15 min after shaking of the microplates for 10 s at medium amplitude. Experiments were repeated to obtain three biological replicates. The mean ODs of the uninoculated media were subtracted from those of the test wells, and the data were transformed by log_10_+1 to obtain a log_10_-adjusted OD for growth curve analysis. Growth rate (μ) and length of the lag phase (hours) were determined from the mean growth curves generated using the GroFit package in R software (51).

### Antibiotic susceptibility assay.

Phenotypic changes in HEPB-adapted *B. vietnamiensis* strain G4 derivatives were characterized by antibiotic susceptibility profiling using Etest strips (bioMérieux, UK) according to manufacturers’ guidelines. Eight antibiotics with various modes of action were examined: amikacin (AMK), azithromycin (AZM), ceftazidime (CAZ), chloramphenicol (CHL), ciprofloxacin (CIP), imipenem (IPM), piperacillin (PIP), and trimethoprim-sulfamethoxazole (SXT).

### Construction of transposon mutants of *B. vietnamiensis* strain G4.

An Escherichia coli S17-1 λ*pir* donor strain carrying pUTmini-Tn*5-luxCDABE*-Km was grown on Luria-Bertani (LB) medium containing 20 μg ml^−1^ kanamycin (KAN) (Sigma) at 37°C. The mini-Tn*5-luxCDABE* transposon was delivered into the recipient *B. vietnamiensis* strain by conjugal mating with the E. coli donor as described by Lewenza et al. (15) with the following modifications. Fresh overnight cultures of recipient and donor were concentrated by centrifugation at 1,600 × *g* for 10 min and resuspended in LB broth containing 10 mM MgSO_4_. Donor and recipient were mixed at a ratio of 1:1 on a nitrocellulose filter (0.2-μm pore size) and incubated on LB agar containing 10 mM MgSO_4_ for 24 h at 37°C. To select *B. vietnamiensis* transconjugants, the mixture was diluted in TSB and inoculated onto TSA containing 30 μg ml^−1^ KAN and 240 units/ml polymyxin B (PMB; Sigma) (PMB was added to counterselect against the E. coli donor). Transconjugants were picked into 96-well plates containing 200 μl TSB and cultured for 24 h on an orbital shaker at 30°C. DMSO was added to achieve a final concentration of 8% (vol/vol), and plates were stored at −80°C.

### Transposon insertion mapping.

Mutant genomic DNA was extracted from overnight cultures using the Maxwell 16 instrument (Promega, Southampton, United Kingdom) and a Maxwell 16 tissue DNA purification kit, according to the manufacturer’s instructions. Genomic DNA flanking the site of transposon insertion was amplified by nested PCR with primers 1 to 4 (Table S7) as described by O’Sullivan et al. (52). The PCR product was sequenced using Primer 3 (Eurofins Genomics, Germany) (Table S7), and interrupted genes were located on the annotated reference *B. vietnamiensis* strain G4 genome (NCBI accession no. GCA_000016205.1).

Bioinformatic analysis was carried out using a virtual machine, hosted by the Cloud Infrastructure for Microbial Bioinformatics (CLIMB) (53). Coding sequence features were extracted (https://github.com/aleimba/bac-genomics-scripts/tree/master/cds_extractor), and a Basic Local Alignment Search Tool (BLAST) algorithm was used to compare nucleotide queries. Additional analysis was performed using the *Burkholderia* Genome Database (54). Twenty mutants from across the mutant bank were selected for validation of single random chromosomal integration of the transposon and sufficient coverage of the multireplicon G4 chromosome. Mutants were confirmed as *B. vietnamiensis* strain G4 by RAPD analysis (50).

### HEPB susceptibility screening of G4 mutants.

*B. vietnamiensis* mutants with altered HEPB susceptibility were identified by agar or broth dilution MIC assays and growth curve analysis as described above. To confirm the phenotype, mutants with altered HEPB susceptibility (relative to that of the wild-type) identified by agar dilution assay in a 96-well plate format were rescreened by broth dilution assay with TSB containing HEPB at the MIC and 0.5× MIC. Mutants with altered susceptibility were selected based on a 20% reduction in OD_600_ in addition to wild-type growth reduction, at both test concentrations. To confirm altered HEPB susceptibility and evaluate fitness, growth curve analysis of the selected mutants was performed in TSB and TSB containing HEPB at half the MIC as described above. Alteration to HEPB susceptibility was defined as equivalent wild-type growth dynamics under control conditions (TSB without HEPB) in conjunction with a lower final OD and/or significantly altered growth rate and longer lag phase. The genetic context of mutants of interest with altered HEPB susceptibility was determined as described above.

### HEPB time-kill assay.

*B. vietnamiensis* strain G4 and P. aeruginosa ATCC 19429 were prepared and cultured (as three biological replicates) as described above for susceptibility testing. Bactericidal activity of HEPB against approximately 1 × 10^6^ CFU was assessed in TSB using 2-fold dilutions of HEPB from 2% (wt/vol) to 0.125% (wt/vol) (2× to 0.5× MIC for strain G4). A 100-μl portion of culture was collected 0, 1, 4, 24 and 168 h postinoculation and diluted in neutralizing solution to inactivate the HEPB, and viable cells were enumerated on TSA using a drop count method.

### Gene expression analysis.

RNA sequence analysis was utilized to determine differential gene expression in response to HEPB, as described by Green et al. (55) with the following modifications. To determine a suitable time point for RNA extraction, growth curve analysis was performed on *B. vietnamiensis* strain G4 cultured in TSB (control) and TSB containing HEPB at 0.75× and 0.5× MIC (test condition) for 24 h as described above. A suitable test concentration and time point were chosen, at which the test and control conditions were at an equivalent growth phase and the cell numbers would yield sufficient RNA for analysis (Fig. S2). Four biological replicates of *B. vietnamiensis* G4 culture (each with four technical replicates) under test and control conditions were harvested. Cells were rapidly cooled using liquid nitrogen and total RNA extracted using the RiboPure RNA purification bacterial kit (Ambion) according to the manufacturer’s guidelines. This included a DNase 1 treatment step to deplete trace amounts of genomic DNA from the total RNA. RNA was quantified using a Qubit fluorometer system and a broad-range RNA kit (Invitrogen). The quality of RNA was determined using a Bioanalyzer with an RNA 6000 Nano kit (Agilent Technologies, Ltd.), according to manufacturer’s recommendations. Total RNA with a high integrity number (≥8) and a ratio of 23S to 16S rRNA of ≥1.5 was concentrated by precipitation to ≥100 ng/μl, and mRNA was enriched using the MICROBExpress bacterial mRNA enrichment kit (Ambion). cDNA library preparations (using the Illumina TruSeq Stranded mRNA kit) and sequencing (on an Illumina NextSeq 500 system) were conducted by the Genomics Research Hub at Cardiff School of Biosciences (https://www.cardiff.ac.uk/biosciences/research/technology-research-hubs/genomics-research).

Bioinformatic analysis was carried out as described by Green et al. (55) with the following modifications. Quality control and adaptor trimming of the paired-end sequencing data were conducted using Trim Galore software v0.4.4 (56). As an additional enrichment step for mRNA sequence data, Artemis software (v16.0) (57) was used to obtain the 16S-to-23S rRNA gene region sequence data from the complete *B. vietnamiensis* strain G4 genome sequence (obtained from NCBI). The RNA-seq reads were first aligned to the 16S-23S rRNA gene region via a Burrows-Wheeler Aligner transformation (BWA) with the BWA-MEM algorithm (v0.7.13-r1126) (58). The aligned sequence reads were removed from the data set using the SAM Tools toolkit (v1.3) (59). The rRNA-clean sequence reads were then aligned to the complete *B. vietnamiensis* strain G4 genome sequence via a BWA using the BWA-MEM algorithm. Sequence Alignment/Map (SAM) files of the aligned sequence reads were then sorted into BAM files using the SAM Tools toolkit. The aligned reads that mapped to annotated gene features of the *B. vietnamiensis* strain G4 genome were counted using the Python program HTSeq-count (v0.6.0) (60). Differential gene expression was determined using the R Bioconductor program DESeq2 (v1.14.1) (61) and was defined as exhibiting a log_2_ fold change of >1.5 as previously described (2, 33, 55).

### Identifying genomic alterations in HEPB-adapted *B. vietnamiensis* G4.

Genomic DNA of the wild-type and HEPB-adapted derivatives (T2s, T2L, and T3) was extracted using the Maxwell 16 tissue DNA purification kit according to manufacturer’s guidelines. DNA library preparations and sequencing on an Illumina NextSeq 500 system were carried out in cooperation with the Genomics Research Hub at Cardiff School of Biosciences. The resulting reads were trimmed using Trim Galore software v0.0.4 and assembled using Unicycler (v0.4.7) (62). Annotation of the assembled draft genomes was conducted using Prokka (v1.12) (63). DNA polymorphisms between the sequence reads of the wild-type genome and the HEPB-adapted derivatives genomes were identified using Snippy (v4.1.0) (64), with default parameters of a minimum base quality of 20, minimum read coverage of 10×, and 90% read concordance at each locus.

### Statistical analysis.

All experiments were performed as 3 biological replicates except for RNA sequencing, which was performed in quadruplicate for each condition. Significant differences (*P* < 0.05) in the mean MICs or MBCs for test groups were determined using a two-sample *t* test for equal or unequal variances as appropriate. Significant differences (*P* < 0.05) in gene expression changes with ≥1.5-fold alteration were determined as described above.

### Data availability.

RNA sequence reads are available in the ArrayExpress database (http://www.ebi.ac.uk/arrayexpress) under accession number E-MTAB-7906. The whole-genome sequence reads of *B. vietnamiensis* strain G4 (wild-type) and HEPB-adapted derivatives T2L, T2s, and T3 are available at the European Nucleotide Archive (accession numbers ERS4125347, ERS4125348, ERS4125349, and ERS4125350, respectively). Gene numbers and nomenclature correlate with those shown on www.burkholderia.com for the *B. vietnamiensis* G4 genome.

## Supplementary Material

Supplemental file 1
