# Supplementary material for: Mapping the Efficacy and Mode of Action of Ethylzingerone [4-(3-Ethoxy-4-Hydroxyphenyl) Butan-2-One] as an Active Agent against Burkholderia Bacteria
Source: Appl Environ Microbiol. 2020 Sep 17;86(19):e01808-20. doi: 10.1128/AEM.01808-20 (PMC7499027; doi:10.1128/AEM.01808-20)
Supplement: Supplemental file 1 [file AEM.01808-20-s0001.pdf]

Mapping the efficacy and mode of action of ethylzingerone [4-(3-ethoxy-4-hydroxyphenyl) butan-2-one] as an active agent against *Burkholderia* bacteria.

Laura Rushton,[a#] Ahmad Khodr,[b*] Florence Menard-Szczebara,[b*] Jean-Yves Maillard,[c] Sylvie Cupferman,[b] and Eshwar Mahenthiralingam [a#]

*: contributed equally

[a] Cardiff School of Biosciences, Cardiff University, Cardiff, UK

[b] International Microbiology Department, L'Oréal Research and Innovation, Chevilly-Larue, France

[c] Cardiff School of Pharmacy and Pharmaceutical Sciences, Cardiff University, Cardiff, UK

Running heading: *Burkholderia* are susceptible to ethylzingerone

[#]**Corresponding author**: Laura Rushton, RushtonL3@cardiff.ac.uk

(ORCID: 0000-0002-4643-6489)

[#]**Co-correspondence:** Eshwar Mahenthiralingam, MahenthiralingamE@cardiff.ac.uk

(ORCID: 0000-0001-9014-3790)

Contents of Supplemental Material

1. **Supplemental Figure S1**. Meta-data analysis showing the anti-*Burkholderia* activity of established preservatives and HEPB at industrially relevant concentrations.
2. **Supplemental Figure S2.** Growth of the model *B. vietnamiensis* strain G4 in control conditions without HEPB, TSB with 4% v/v DMSO and in TSB test culture with up to 0.395 % w/v HEPB
3. **Supplemental Figure S3**. Confocal microscopy images of *B. vietnamiensis* strain G4 cultured in the presence and absence of HEPB**.**
4. **Supplemental Table S1**. *Burkholderia* strains used in this study.
5. **Supplemental Table S2**. Non-*Burkholderia* strains used in this study.
6. **Supplemental Table S3**. The minimum inhibitory concentration and minimum bactericidal concentration of HEPB for 58 *Burkholderia* strains evaluated in this study.
7. **Supplemental Table S4**. The minimum inhibitory concentration and minimum bactericidal concentration of HEPB for 7 non-*Burkholderia* strains evaluated in this study.
8. **Supplemental Table S5**. Significantly Up-regulated genes (≥ 1.5-fold change) of *B. vietnamiensis* strain G4 in response to sub-inhibitory (0.5 x MIC) of HEPB.
9. **Supplemental Table S6**. Significantly down-regulated genes (≥ 1.5-fold change) of *B. vietnamiensis* strain G4 in response to sub-inhibitory (0.5 x MIC) of HEPB.
10. **Supplemental Table S7**. PCR primers used in this study.
11. **Supplemental References**

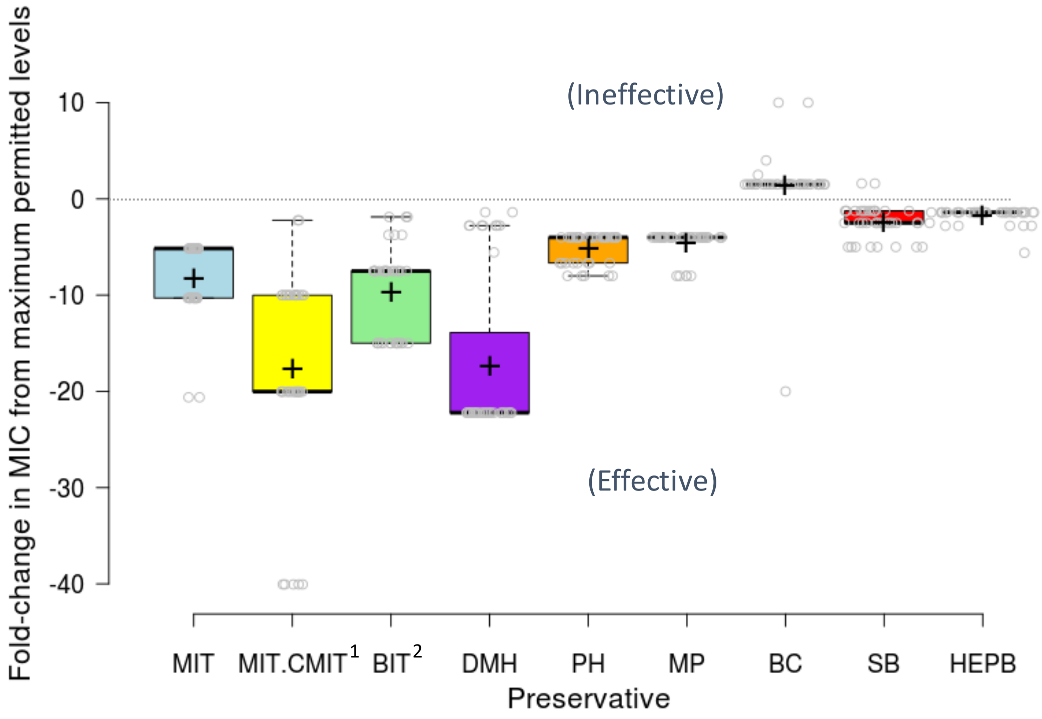

Supplemental Figure S1. The anti-*Burkholderia* activity of established preservatives and HEPB at industrially relevant concentrations. Fold-change in the MICs of eight preservatives and HEPB for 39 *Burkholderia* strains, from the maximum preservative levels permitted in personal care products. Centre lines show the medians; box limits indicate the 25th and 75th percentiles as determined by R software; whiskers extend 1.5 times the interquartile range from the 25th and 75th percentiles, outliers are represented by dots; crosses represent sample means; data points are plotted as open circles. n = 39 sample points. MIC, minimum inhibitory concentration. MIT, methylisothiazolinone; MIT.CMIT, methylisothiazolinone and chloromethylisothiazolinone; BIT, benzisothiazolinone; PH, phenoxyethanol; BC, benzethonium chloride; DMH, dimethylol dimethyl hydantoin; SB, sodium benzoate; MP, methyl paraben; and HEPB, [4-(3-ethoxy-4-hydroxyphenyl) butan-2-one] . [1] A cosmetic grade commercial blend evaluated. [2] Not permitted for use in EU regulated countries, manufacturers recommended level taken as maximum. The maximum active level for use in rinse off personal care products according to EU Regulation No 1223/2009 Annex V(1) : MIT, 0.0015% ; MIT.CMIT, 0.0015%;BC, 0.1%; DMH, 0.6%; PH, 1%; MP, 0.4%; SB, 0.5%; HEPB, 0.7%. Manufacturers recommended maximum use level of BIT set at 0.015% active. BCC, Cardiff strain collection. MIC of HEPB determined by dilution assay on tryptic soya agar (this study). All other MIC values were determined by agar dilution assay on a minimal Basal Salts medium (Rushton *et al.* 2013).

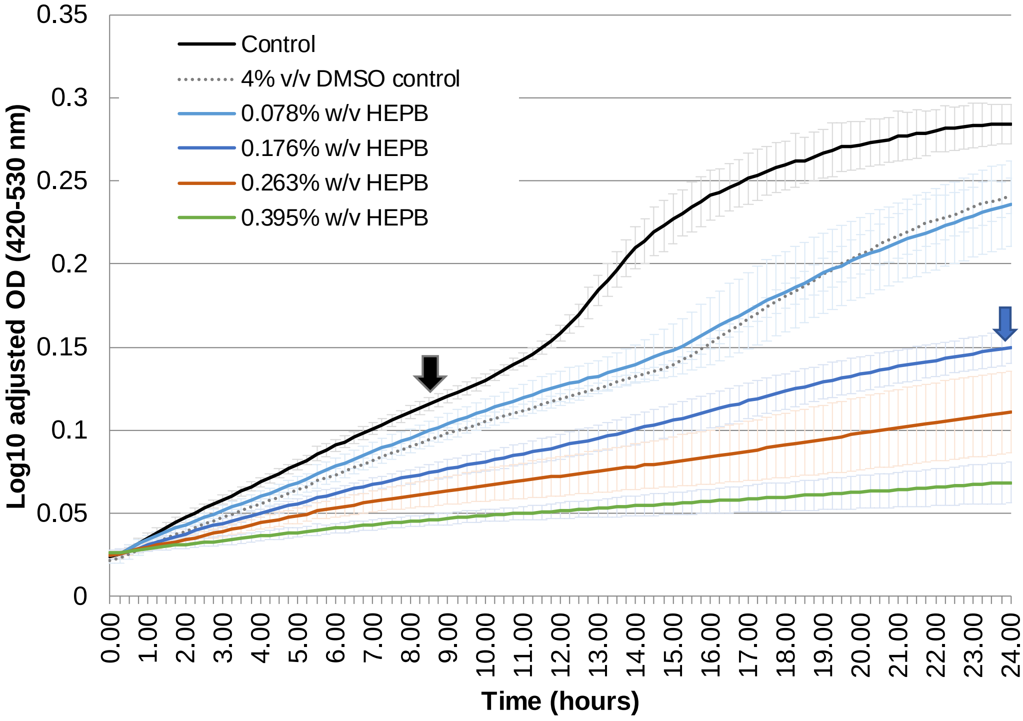

Supplemental Figure S2. Growth of the model *B. vietnamiensis* strain G4 in control conditions without HEPB, TSB with 4% v/v DMSO and in TSB test culture with up to 0.395 % w/v HEPB. The MIC (0.395% w/v) was defined as an 80% reduction in optical density from the control at 24 hours. Each symbol indicated the means ± STDEV for three biological replicates. Arrows indicate sampling times (OD 0.3 - 0.4) for gene expression analysis

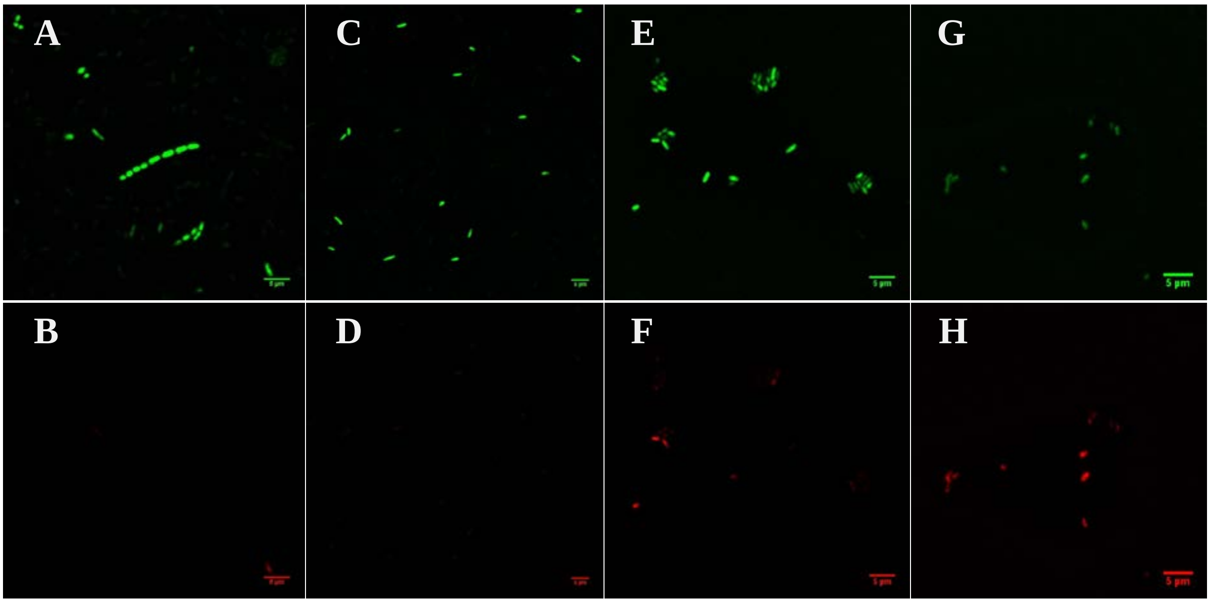

Supplemental Figure S3. Confocal microscopy images of *B. vietnamiensis* strain G4 cultured in the presence and absence of HEPB. (A-B) control, and HEPB at (C-D) 0.25% w/v, (E-F) 0.5% w/v, (G-H) 1% w/v. Cells were analysed after incubation with BacLITE live (green)/dead (red) stain containing SYT09 (green) and propidium iodide (red) for 10 min and imaged using a Zeiss LSM880 Airyscan confocal microscope. The bacterial cells shown here are viewed as split channels (red/green) and are representative of the morphotypes and cell aggregates observed in the numerous fields examined. Uptake of propidium iodide (red), and fragmentation of cells, increased with increasing concentrations of HEPB.

Supplemental Table S1. *Burkholderia* strains used in this study

Species & strain name	Accession number from BCCM/LMG culture collection (Other strain designations)	Isolation source and other information	Isolation source code	MLST Sequence type
B. ambifaria				
AMMD [ESP a]	LMG 19182 [T]	Pea rhizosphere, USA; genome available (http://genome.jgi-psf.org/mic_cur1.html)	ENV	77
BCC0267 [ESP a]	LMG 19467	Cystic fibrosis patient	CLIN	78
BCC0338 [a]	LMG 17828	Corn rhizosphere, USA	ENV	74
B. anthina				
BCC0635 [ESP a]	LMG 16670	Carludovica palmata, rhizosphere, UK.	ENV	89
BCC0639 [ESP a]	LMG 20980 [T]	Soil, USA	ENV	86
BCC0485 [ESP]	LMG21821	Cystic fibrosis patient, USA	CLIN	90
B. arboris				
BCC0049 [a]	-	Clinical isolate, Europe	CLIN	110
BCC1306 [a]	-	Environmental industrial	ENVI	325
BCC1310 [a]	-	Environmental Industrial	ENVI	327
B. cenocepacia III-A				
BCC0018 [ESP a]	LMG 16659	Cystic fibrosis patient ,UK	CLIN	35
J2315 [ESP]	LMG 16656 [T]	Cystic fibrosis patient sputum, UK; genome available (www.sanger.ac.uk/Projects/B_cenocepacia/)	CLIN	28
B. cenocepacia III-B				
HI2424 [a]	-	Soil, USA; genome available (http://genome.jgi-psf.org/mic_cur1.html)	ENV	122
B. cepacia				
BCC0001 [ESP a]	LMG 1222 [T]	Onion rot, USA	ENV	10
BCC0002 [ESP a]	LMG 2161	Forest soil, Trinidad	ENV	1
BCC0003 [ESP a]	LMG 18821	Cystic fibrosis patient, Australia	CLIN	5
B. contaminans				
SAR-1 [a]	LMG 23255	Cystic fibrosis patient, Czech Republic; metagenomic strain from Sargasso sea	CLIN	102
BCC1315 [a]	-	Environmental industrial	ENVI	341
BCC0339	-	Cystic fibrosis patient	CLIN	404
B. diffusa				
BCC0106 [a]	LMG 24266	Cystic fibrosis patient's throat, Canada	CLIN	107
BCC0169 [a]	(ATCC 29352)	Soil	ENV	108
AU1075 [a]	LMG 24065	Cystic fibrosis patient, USA	CLIN	164

B. dolosa				
BCC0161	FC0380	Cystic fibrosis patient	CLIN	71
AU0645 [ESP a]	LMG 18943 [T]	Cystic fibrosis patient, USA	CLIN	72
AU3556 [a]	-	Cystic fibrosis patient	CLIN	215
B. lata				
Strain 383 [a]	LMG 22485 [T]	Forest soil, Trinidad; genome available (http://genome.jgi-psf.org/bur94/bur94.home.html)	ENV	101
BCC1296 [a]	-	Environmental industrial	ENVI	119
BCC1406 [a]	-	Environmental industrial	ENVI	103
B. latens				
BCC1625	LMG24064[T]	Cystic fibrosis patient, Italy	CLIN	238
BCC1626	LMG24264	Cystic fibrosis patient, UK	CLIN	1022
BCC1892	LMG24265	Cystic fibrosis patient, UK	CLIN	1545
B. metallica				
BCC0095 [a]	-	Cystic fibrosis patient sputum, Canada	CLIN	288
AU0553 [a]	LMG 24068 [T]	Cystic fibrosis patient, USA	CLIN	511
B. multivorans				
BCC0149		Cystic fibrosis patient	CLIN	2
ATCC17616 [ESP a]	LMG 17588	Soil, US ; genome available (http://genome.jgi-psf.org/mic_cur1.html)	ENV	21
BCC1560 [a]	-	Environmental industrial	ENVI	439
B. pseudomultivorans				
BCC1894	LMG 26883	Cystic fibrosis patient sputum,USA	CLIN	536
BCC1191 [a]	D1443	Cystic fibrosis patient sputum	CLIN	307
B. pyrrocinia				
BCC0171 [ESP a]	LMG 21822	Corn field soil, USA	ENV	95
BCC0180 [T ESP a]	LMG 14191 [T]	Soil	ENV	41
BCC0476 [ESP a]	LMG 21823	Water, UK	ENV	92
B. seminalis				
BCC1627 [T]	LMG 24067	Cystic fibrosis patient, USA	CLIN	473
BCC1628	LMG 19587	Oryza sativa seed, Philippines	ENV	383
BCC1893	LMG 24272	Nosocomial infection, Thailand	CLIN	1557
B. stabilis				
BCC0023 [ESP a]	LMG 14294[T]	Cystic fibrosis patient sputum, Belgium	CLIN	50
BCC0286 [a]	(ATCC 35254)	Povidone-iodine solution, USA	ENV	51
AU6735 [a]	-	Cystic fibrosis patient	CLIN	53

B. stagnalis				
BCC1350	HI3541	Soil, USA	ENV	865
BCC1887 [T]	LMG 28156	Soil, Australia	ENV	787
BCC1896	LMG 28157	Soil, Australia	ENV	789
B. territorii				
BC1888 [T]	LMG 28158	Water, Australia	ENV	791
BCC1897	LMG 28159	Water, Australia	ENV	794
B. ubonensis				
BCC1603 [a]	LMG 20358	Surface soil, Thailand	ENV	299
B. vietnamiensis				
BCC0028 [ESP a]	LMG 16232	Cystic fibrosis patient sputum, Sweden	CLIN	200
BCC0195	LMG 22486,G4	Wastewater, USA	ENV	60
BCC1309 [a]	-	Environmental industrial	ENVI	326
B. gladioli				
BCC1317 [a]		Environmental industrial	ENVI	949
BCC0238	MA4	Cystic Fibrosis patient	CLIN	946
BCC0507	PF(#428)	Cystic Fibrosis patient	CLIN	948
B. plantarii				
BCC0777	LMG 9035	Oryza sativa seedling with blight, Japan	ENV	1024

BCCM/LMG, Belgian co-ordinated collections of micro-organisms, Ghent.

ATCC, American type culture collection BCC.

Cardiff strain collection CLIN, clinical; ENV, environmental; ENVI, environmental industrial.

MLST, Multi locus sequence typing.

[a] Strain preservative susceptibility profiled by Rushton *et al.* (2).

[T] Type strain.

[ESP] *Burkholderia cepacia* complex experimental strain panel.

Supplemental Table S2. Non-*Burkholderia* strains used in this study.

**Species, Accession number**	**Isolation source**	**Isolation source code**
Paraburkholderia fungorum		
LMG 16225 [T]	Cystic Fibrosis patient	CLIN
Paraburkholderia graminis		
LMG 18924	Maize, France	ENV
Staphylococcus aureus		
ATCC 25923 [A]	Human lesion	CLIN
Staphylococcus aureus		
NCIMB 9518 [B]	Clinical	CLIN
Pseudomonas aeruginosa		
ATCC 27853	Blood culture	CLIN
Pseudomonas aeruginosa		
ATCC 19429 [A]	Urine	CLIN
Escherichia coli		
ATCC 8739 [A]	Faeces	CLIN

LMG, Belgian co-ordinated collections of micro-organisms, Ghent.

ATCC, American type culture collection BCC.

Cardiff strain collection CLIN, clinical; ENV, environmental.

[T] Type strain.

[A] Reference strain for assay of antimicrobial activity of preservatives.

[B] Reference strain for antibiotic susceptibility testing.

Supplemental Table S3. The minimum inhibitory concentration and minimum bactericidal concentration of HEPB for 58 *Burkholderia* strains evaluated in this study

		**HEPB Minimum inhibitory concentration (% w/v)**						**HEPB Minimum bactericidal concentration (% w/v)**					
**Species**	**Strain ID**	**Biological replicate**			**Median**	**Mean (± *STDEV*)**		**Biological replicate**			**Median**	**Mean (± *STDEV*)**	
		1	***2***	***3***				***1***	***2***	***3***			
Burkholderia ambifaria	AMMD	0.5	0.5	0.5	0.5	**0.5**	*0.00*	1	1	1	1	**1**	*0.0*
	BCC0267	0.125	0.125	0.125	0.125	**0.125**	*0.00*	0.5	0.5	0.5	0.5	**0.5**	*0.0*
	BCC0338	0.5	0.5	0.5	0.5	**0.5**	*0.00*	1	1	1	1	**1**	*0.0*
Burkholderia anthina	BCC0635	0.5	0.5	0.5	0.5	**0.5**	*0.00*	1	1	1	1	**1**	*0.0*
	BCC0639	0.5	0.25	0.5	0.5	**0.42**	*0.14*	1	1	1	1	**1**	*0.0*
	BCC0485	0.25	0.25	0.25	0.25	**0.25**	*0.00*	1	1	0.5	1	**0.8**	*0.3*
Burkholderia arboris	BCC1310	0.25	0.25	0.0625	0.25	**0.19**	*0.11*	1	1	1	1	**1**	*0.0*
	BCC0049	0.25	0.25	N/A	0.25	**0.25**	*0.00*	0.5	0.5	0.5	0.5	**0.5**	*0.0*
	BCC1306	0.5	0.5	0.5	0.5	**0.5**	*0.00*	0.5	1	1	1	**0.8**	*0.3*
Burkholderia cenocepacia III-A	BCC0018	0.5	0.5	0.5	0.5	**0.5**	*0.00*	1	1	1	1	**1**	*0.0*
Burkholderia cenocepacia III-B	HI242	0.5	0.5	0.5	0.5	**0.5**	*0.00*	1	1	1	1	**1**	*0.0*
Burkholderia cenocepacia III-A	J2315	0.5	0.5	0.5	0.5	**0.5**	*0.00*	1	0.5	0.5	0.5	**0.7**	*0.3*
Burkholderia cepacia	BCC0001	0.5	0.5	0.5	0.5	**0.5**	*0.00*	1	1	1	1	**1**	*0.0*
	BCC0002	0.5	0.5	0.5	0.5	**0.5**	*0.00*	1	1	1	1	**1**	*0.0*
	BCC0003	0.5	0.5	0.5	0.5	**0.5**	*0.00*	1	0.5	0.5	0.5	**0.7**	*0.3*

Burkholderia contaminans	SAR-1	0.5	0.25	0.5	0.5	**0.42**	*0.14*	1	0.5	0.5	0.5	**0.7**	*0.3*
	BCC1315	0.5	0.5	0.5	0.5	**0.5**	*0.00*	1	1	1	1	**1**	*0.0*
	BCC339	0.5	0.5	0.5	0.5	**0.5**	*0.00*	0.5	1	1	1	**0.8**	*0.3*
Burkholderia diffusa	BCC0169	0.5	0.5	0.5	0.5	**0.5**	*0.00*	1	1	1	1	**1**	*0.0*
	BCC0106	0.5	0.5	0.5	0.5	**0.5**	*0.00*	1	1	1	1	**1**	*0.0*
	AU1075	0.5	0.25	0.5	0.5	**0.42**	*0.14*	1	0.5	1	1	**0.8**	*0.3*
Burkholderia dolosa	AU3556	0.25	0.25	0.25	0.25	**0.25**	*0.00*	1	1	0.5	1	**0.8**	*0.3*
	BCC0161	0.5	0.25	0.5	0.5	**0.42**	*0.14*	1	0.5	0.5	0.5	**0.7**	*0.3*
	AU0645	0.5	0.5	0.5	0.5	**0.5**	*0.00*	1	1	0.5	1	**0.8**	*0.3*
Burkholderia lata	383	0.5	0.5	0.5	0.5	**0.5**	*0.00*	1	1	1	1	**1**	*0.0*
	BCC1296	0.5	0.5	N/A	0.5	**0.5**	*0.00*	1	1	1	1	**1**	*0.0*
	BCC1406	0.5	0.5	0.5	0.5	**0.5**	*0.00*	1	1	1	1	**1**	*0.0*
Burkholderia latens	BCC1625	0.5	0.5	0.5	0.5	**0.5**	*0.00*	1	1	1	1	**1**	*0.0*
	BCC1626	0.5	0.5	0.5	0.5	**0.5**	*0.00*	1	1	0.5	1	**0.8**	*0.3*
	BCC1892	0.5	0.5	0.5	0.5	**0.5**	*0.00*	1	1	0.5	1	**0.8**	*0.3*
Burkholderia metallica	BCC0095	0.5	0.5	0.5	0.5	**0.5**	*0.00*	1	0.5	1	1	**0.8**	*0.3*
	AU0553	0.5	0.5	0.5	0.5	**0.5**	*0.00*	1	0.5	1	1	**0.8**	*0.3*
Burkholderia multivorans	ATCC 17616	0.5	0.5	0.5	0.5	**0.5**	*0.00*	1	1	1	1	**1**	*0.0*
	BCC1560	0.5	0.5	0.5	0.5	**0.5**	*0.00*	2	1	0.5	1	**1.2**	*0.8*
Burkholderia pseudomultivorans	BCC1894	0.5	0.5	0.5	0.5	**0.5**	*0.00*	1	0.5	0.5	0.5	**0.7**	*0.3*
	BCC1191	0.5	0.5	0.5	0.5	**0.5**	*0.00*	1	0.5	0.5	0.5	**0.7**	*0.3*
Burkholderia pyrrocinia	BCC0171	0.5	0.5	0.5	0.5	**0.5**	*0.00*	1	1	1	1	**1**	*0.0*
	BCC0180	0.5	0.5	0.5	0.5	**0.5**	*0.00*	1	1	1	1	**1**	*0.0*
	BCC0476	0.5	0.5	0.5	0.5	**0.5**	*0.00*	0.5	0.5	0.5	0.5	**0.5**	*0.0*
Burkholderia seminalis	BCC1627	0.5	0.5	0.5	0.5	**0.5**	*0.00*	1	1	1	1	**1**	*0.0*
	BCC1628	0.5	0.5	0.5	0.5	**0.5**	*0.00*	1	1	1	1	**1**	*0.0*
	BCC1893	0.5	0.5	0.5	0.5	**0.5**	*0.00*	2	1	1	1	**1.3**	*0.6*

Burkholderia stabilis	BCC0023	0.5	0.5	0.5	0.5	**0.5**	*0.00*	1	1	1	1	**1**	*0.0*
	BCC0286	0.5	0.5	0.5	0.5	**0.5**	*0.00*	2	1	1	1	**1.3**	*0.6*
	AU6735	0.5	0.5	1	0.5	**0.67**	*0.29*	1	1	1	1	**1**	*0.0*
Burkholderia stagnalis	BCC1350	0.5	0.5	0.5	0.5	**0.5**	*0.00*	1	0.5	1	1	**0.8**	*0.3*
	BCC1887	0.5	0.5	0.5	0.5	**0.5**	*0.00*	1	1	1	1	**1**	*0.0*
	BCC1896	0.5	0.5	0.5	0.5	**0.5**	*0.00*	1	1	1	1	**1**	*0.0*
Burkholderia territorii	BCC1888	0.5	0.5	0.5	0.5	**0.5**	*0.00*	1	1	1	1	**1**	*0.0*
	BCC1897	0.5	0.5	0.5	0.5	**0.5**	*0.00*	2	1	1	1	**1.3**	*0.6*
Burkholderia ubonensis	BCC1603	0.5	0.5	0.5	0.5	**0.5**	*0.00*	1	1	0.5	1	**0.8**	*0.3*
Burkholderia vietnamiensis	BCC0195	0.25	0.25	0.25	0.25	**0.25**	*0.00*	0.5	0.5	0.5	0.5	**0.5**	*0.0*
	BCC0028	0.5	0.5	0.5	0.5	**0.5**	*0.00*	2	1	1	1	**1.3**	*0.6*
	BCC1309	0.25	0.25	0.25	0.25	**0.25**	*0.00*	0.5	1	0.5	0.5	**0.7**	*0.3*
Burkholderia plantarii	BCC0777	0.25	0.25	0.25	0.25	**0.25**	*0.00*	0.5	0.5	0.5	0.5	**0.5**	*0.0*
Burkholderia gladioli	BCC1317	0.25	0.25	0.5	0.25	**0.333**	*0.14*	2	0.5	0.5	0.5	**1**	*0.9*
	BCC0238	0.25	0.5	0.25	0.25	**0.333**	*0.14*	0.5	0.5	0.5	0.5	**0.5**	*0.0*
	BCC0507	0.5	0.125	0.5	0.5	**0.375**	*0.22*	1	1	1	1	**1**	*0.0*

Supplemental Table S4. The minimum inhibitory concentration and minimum bactericidal concentration of HEPB for 7 non-*Burkholderia* strains evaluated in this study

		**HEPB Minimum inhibitory concentration (% w/v)**						**HEPB Minimum bactericidal concentration (% w/v)**					
**Species**	**Strain ID**	**Biological replicate**			**Median**	**Mean (± *STDEV*)**		**Biological replicate**			**Median**	**Mean (± *STDEV*)**	
		1	***2***	***3***				***1***	***2***	***3***			
Paraburkholderia fungorum	LMG 16225	0.125	0.125	0.125	0.1	**0.13**	*0.00*	0.5	0.5	0.5	0.5	**0.5**	*0.0*
Paraburkholderia graminis	LMG 18924	0.125	0.125	0.125	0.1	**0.13**	*0.00*	1	N/A	N/A	1.0	**1.0**	*0.0*
Staphylococcus	ATCC 25923	1	1	1	1.0	**1.00**	*0.00*	>2	>2	>2	N/A	**N/A**	*N/A*
aureus	NCIMB 9518	1	1	1	1.0	**1.00**	*0.00*	2	>2	1	1.5	**1.5**	*0.7*
Pseudomonas	ATCC 27853	>2	>2	>1	N/A	**>2**	*N/A*	>2	>2	>2	N/A	**N/A**	*N/A*
aeruginosa	ATCC 1929	>2	1	>1	1.0	**>2**	*N/A*	>2	>2	>2	N/A	**N/A**	*N/A*
Escherichia coli	ATCC 8739	1	1	1	1.0	**1.00**	*0.00*	>2	>2	>2	N/A	**N/A**	*N/A*

Supplemental Table S5. Significantly Up-regulated genes (≥ 1.5-fold change) of *B. vietnamiensis* strain G4 in response to sub-inhibitory (0.5 x MIC) of HEPB.

Gene ID	Replicon	Log2 Fold Change	Adjusted p-value	Putative Gene function [1]	COG Category [2]
**Bcep1808_2712** [d]	chr 1	4.26	7.63E-30	sorbitol dehydrogenase (short-chain dehydrogenase reductase)	Function unknown
**Bcep1808_2707** [c]	chr 1	4.06	1.06E-11	binding-protein-dependent transport systems inner membrane component	Carbohydrate transport and metabolism
**Bcep1808_7230**	pBVIE02	3.84	1.84E-13	resolvase domain-containing protein	Replication, recombination and repair
**Bcep1808_2709**	chr 1	3.82	1.02E-30	extracellular solute-binding protein	Carbohydrate transport and metabolism
**Bcep1808_2710** [d]	chr 1	3.56	9.74E-08	tagatose-bisphosphate aldolase noncatalytic subunit	Carbohydrate transport and metabolism
**Bcep1808_2705** [c]	chr 1	3.54	6.11E-10	ABC transporter-like protein	Carbohydrate transport and metabolism
**Bcep1808_7052** [o]	pBVIE02	3.39	7.36E-11	cobyrinic acid a,c-diamide synthase	Cell cycle control, cell division, chromosome partitioning
**Bcep1808_0270** [b]	chr 1	3.35	5.21E-79	hypothetical protein	Function unknown
**Bcep1808_0269** [b]	chr 1	3.34	1.55E-75	hypothetical protein	Function unknown
**Bcep1808_2708** [c]	chr 1	3.32	2.63E-11	binding-protein-dependent transport systems inner membrane component	Carbohydrate transport and metabolism

**Bcep1808_7053** [o]	pBVIE02	3.24	1.74E-11	parB-like partition protein	Transcription
**Bcep1808_7139**	pBVIE02	3.21	9.79E-18	phage integrase family protein	Replication, recombination and repair
**Bcep1808_0268** [b]	chr 1	3.16	1.53E-84	UBA/THIF-type NAD/FAD binding protein	Coenzyme transport and metabolism
**Bcep1808_7203** [q]	pBVIE02	3.05	4.04E-08	phage integrase family protein	Replication, recombination and repair
**Bcep1808_7040**	pBVIE02	2.92	2.41E-08	hypothetical protein	Function unknown
**Bcep1808_2711** [d]	chr 1	2.85	2.50E-05	ribokinase-like domain-containing protein	Carbohydrate transport and metabolism
**Bcep1808_7379**	pBVIE03	2.78	8.81E-29	hypothetical protein	Function unknown
**Bcep1808_7204** [q]	pBVIE02	2.67	1.68E-14	hypothetical protein	Function unknown
**Bcep1808_6222**	chr 3	2.65	9.21E-54	hypothetical protein	Function unknown
**Bcep1808_7286**	pBVIE02	2.58	2.77E-13	hypothetical protein	Function unknown
**Bcep1808_7261**	pBVIE02	2.57	4.79E-10	MerR family transcriptional regulator	Transcription
**Bcep1808_7051** [o]	pBVIE02	2.51	9.33E-05	hypothetical protein	Function unknown
**Bcep1808_7049**	pBVIE02	2.48	2.43E-04	hypothetical protein	Function unknown
**Bcep1808_7293**	pBVIE02	2.42	5.98E-05	ModE family transcriptional regulator	General function prediction only
**Bcep1808_7279**	pBVIE02	2.37	4.94E-08	hypothetical protein	Function unknown
**Bcep1808_6165** [j]	chr 3	2.36	3.62E-11	hypothetical protein	Function unknown
**Bcep1808_7294**	pBVIE02	2.34	3.01E-11	tungstate/molybdate binding protein	Inorganic ion transport and metabolism
**Bcep1808_6148**	chr 3	2.31	2.07E-07	hypothetical protein	Function unknown
**Bcep1808_7054**	pBVIE02	2.30	1.24E-05	hypothetical protein	Replication, recombination and repair
**Bcep1808_7146**	pBVIE02	2.26	4.48E-04	hypothetical protein	Function unknown

**Bcep1808_7205** [q]	pBVIE02	2.26	1.00E-05	TnpC, transposase	Replication, recombination and repair
**Bcep1808_6152**	chr 3	2.23	4.95E-08	two component LuxR family transcriptional regulator	Signal transduction mechanisms
**Bcep1808_6181** [m]	chr 3	2.23	4.57E-05	BadM/Rrf2 family transcriptional regulator	Transcription
**Bcep1808_6177** [l]	chr 3	2.22	1.07E-06	enoyl-(acyl carrier protein) reductase	Lipid transport and metabolism
**Bcep1808_6188** [n]	chr 3	2.21	2.22E-08	alkylhydroperoxidase	Function unknown
**Bcep1808_6189**	chr 3	2.18	2.67E-04	hypothetical protein	Function unknown
**Bcep1808_3537**	chr 2	2.18	1.14E-29	5- methyltetrahydropteroyltriglutamate/homocysteine S-methyltransferase	Amino acid transport and metabolism
**Bcep1808_7237** [r]	pBVIE02	2.17	5.45E-08	integrase catalytic subunit	Replication, recombination and repair
**Bcep1808_7198** [p]	pBVIE02	2.15	2.28E-03	TniB family protein	Function unknown
**Bcep1808_5376**	chr 2	2.15	2.44E-08	XRE family transcriptional regulator	Transcription
**Bcep1808_6183** [m]	chr 3	2.10	1.47E-06	hemerythrin HHE cation binding domain-containing protein	Function unknown
**Bcep1808_6154** [h]	chr 3	2.10	2.66E-05	cytochrome c, class I	Energy production and conversion
**Bcep1808_7281**	pBVIE02	2.10	9.77E-14	hypothetical protein	Function unknown
**Bcep1808_7282**	pBVIE02	2.10	2.21E-09	hypothetical protein	General function prediction only
**Bcep1808_7283**	pBVIE02	2.10	7.83E-06	LemA family protein	Function unknown
**Bcep1808_2724**	chr 1	2.09	1.83E-08	TetR family transcriptional regulator	Transcription
**Bcep1808_7264**	pBVIE02	2.08	5.73E-14	multicopper oxidase, type 2	Secondary metabolites biosynthesis, transport and catabolism
**Bcep1808_6174**	chr 3	2.06	7.27E-04	hypothetical protein	Function unknown
**Bcep1808_6195**	chr 3	2.05	1.86E-06	transposase Tn3 family protein	Replication, recombination and repair

**Bcep1808_6153** [h]	chr 3	2.03	3.63E-09	cytochrome c4	Energy production and conversion
**Bcep1808_6190**	chr 3	2.02	3.29E-05	hypothetical protein	Function unknown
**Bcep1808_7197** [p]	pBVIE02	2.01	1.12E-05	integrase catalytic subunit	Function unknown
**Bcep1808_7155**	pBVIE02	2.01	8.15E-03	transposase, IS4 family protein	Replication, recombination and repair
**Bcep1808_0171** [a]	chr 1	1.99	2.32E-45	hypothetical protein	Function unknown
**Bcep1808_6175** [l]	chr 3	1.98	1.73E-07	phosphate acetyltransferase	Energy production and conversion
**Bcep1808_1155**	chr 1	1.98	9.21E-04	hypothetical protein	Function unknown
**Bcep1808_7750**	pBVIE05	1.98	5.79E-36	hypothetical protein	Function unknown
**Bcep1808_6176** [l]	chr 3	1.98	4.82E-05	acetate kinase	Energy production and conversion
**Bcep1808_6156** [i]	chr 3	1.97	1.62E-03	cytochrome c oxidase, subunit II	Energy production and conversion
**Bcep1808_7143**	pBVIE02	1.97	7.73E-04	hypothetical protein	Function unknown
**Bcep1808_7171**	pBVIE02	1.96	5.51E-05	transposase Tn3 family protein	Replication, recombination and repair
**Bcep1808_7751**	pBVIE05	1.96	6.29E-31	hypothetical protein	Function unknown
**Bcep1808_7445**	pBVIE03	1.95	5.37E-15	MazE family transcriptional regulator	Signal transduction mechanisms
**Bcep1808_6304**	chr 3	1.95	1.55E-02	hypothetical protein	Function unknown
**Bcep1808_7242**	pBVIE02	1.94	7.56E-10	hypothetical protein	Function unknown
**Bcep1808_7183**	pBVIE02	1.94	5.04E-03	MerR family transcriptional regulator	Transcription
**Bcep1808_1216**	chr 1	1.92	7.18E-03	integral membrane protein TerC	Inorganic ion transport and metabolism
**Bcep1808_7330**	pBVIE03	1.92	5.75E-04	hypothetical protein	Function unknown
**Bcep1808_7160**	pBVIE02	1.92	6.65E-03	integrase catalytic subunit	Function unknown

**Bcep1808_7278**	pBVIE02	1.91	1.01E-05	hypothetical protein	Posttranslational modification, protein turnover, chaperones
**Bcep1808_2491**	chr 1	1.91	1.00E-21	hypothetical protein	Function unknown
**Bcep1808_7266**	pBVIE02	1.91	4.10E-04	S-adenosylmethionine synthetase	Amino acid transport and metabolism
**Bcep1808_7043**	pBVIE02	1.91	2.26E-03	integrase catalytic subunit	Replication, recombination and repair
**Bcep1808_5377**	chr 2	1.90	5.06E-05	FAD dependent oxidoreductase	Amino acid transport and metabolism
**Bcep1808_6170** [k]	chr 3	1.89	1.22E-05	secretion protein HlyD family protein	Defence mechanisms
**Bcep1808_6180**	chr 3	1.89	4.25E-06	phage integrase family protein	Replication, recombination and repair
**Bcep1808_6178**	chr 3	1.89	3.61E-04	transposase, IS4 family protein	Function unknown
**Bcep1808_6166** [j]	chr 3	1.88	8.58E-03	acetate kinase	Energy production and conversion
**Bcep1808_7048**	pBVIE02	1.87	1.16E-04	hypothetical protein	Function unknown
**Bcep1808_7271**	pBVIE02	1.87	6.42E-04	2-octaprenylphenol hydroxylase	General function prediction only
**Bcep1808_6338**	chr 3	1.87	3.39E-08	Crp/FNR family transcriptional regulator	Signal transduction mechanisms
**Bcep1808_6193**	chr 3	1.86	2.28E-06	FAD-dependent pyridine nucleotide-disulphide oxidoreductase	Posttranslational modification, protein turnover, chaperones
**Bcep1808_6120** [g]	chr 3	1.86	1.80E-33	hypothetical protein	Function unknown
**Bcep1808_7277**	pBVIE02	1.85	2.59E-08	FtsH-2 peptidase	Posttranslational modification, protein turnover, chaperones
**Bcep1808_6203**	chr 3	1.85	7.45E-06	two component LuxR family transcriptional regulator	Signal transduction mechanisms
**Bcep1808_6210**	chr 3	1.84	2.33E-05	transport-associated	Function unknown
**Bcep1808_6169**	chr 3	1.83	4.10E-04	AraC family transcriptional regulator	Transcription
**Bcep1808_7276**	pBVIE02	1.83	1.74E-03	hypothetical protein	Function unknown

**Bcep1808_7748**	pBVIE05	1.82	2.51E-02	hypothetical protein	Function unknown
**Bcep1808_0279**	chr 1	1.82	2.06E-27	phage integrase family protein	Replication, recombination and repair
**Bcep1808_2702**	chr 1	1.82	1.04E-15	mannitol dehydrogenase domain-containing protein	Carbohydrate transport and metabolism
**Bcep1808_4656**	chr 2	1.81	2.16E-04	hypothetical protein	Transcription
**Bcep1808_7251**	pBVIE02	1.80	1.26E-04	hypothetical protein	Function unknown
**Bcep1808_1553**	chr 1	1.80	6.15E-09	sulphate ABC transporter periplasmic sulphate-binding protein	Inorganic ion transport and metabolism
**Bcep1808_3982**	chr 2	1.79	1.60E-05	LemA family protein	Function unknown
**Bcep1808_7252**	pBVIE02	1.79	1.41E-03	hypothetical protein	Function unknown
**Bcep1808_7288**	pBVIE02	1.79	1.64E-05	transposase Tn3 family protein	Replication, recombination and repair
**Bcep1808_6182** [m]	chr 3	1.79	7.22E-05	NnrS family protein	Inorganic ion transport and metabolism
**Bcep1808_7267**	pBVIE02	1.79	2.59E-04	hypothetical protein	Lipid transport and metabolism
**Bcep1808_6155** [h]	chr 3	1.78	2.80E-04	hypothetical protein	Function unknown
**Bcep1808_7239**	pBVIE02	1.78	4.81E-04	hypothetical protein	Function unknown
**Bcep1808_7292**	pBVIE02	1.78	8.10E-03	hypothetical protein	Function unknown
**Bcep1808_6150**	chr 3	1.77	8.25E-06	histone family protein nucleoid-structuring protein H-NS	General function prediction only
**Bcep1808_7285**	pBVIE02	1.77	2.88E-03	YHS domain-containing protein	Function unknown
**Bcep1808_7263**	pBVIE02	1.77	6.86E-04	hypothetical protein	Function unknown

**Bcep1808_6268**	chr 3	1.76	3.48E-02	alpha/beta hydrolase domain-containing protein	Inorganic ion transport and metabolism
**Bcep1808_7749**	pBVIE05	1.76	1.60E-15	hypothetical protein	Function unknown
**Bcep1808_6184** [m]	chr 3	1.76	1.05E-03	hypothetical protein	Function unknown
**Bcep1808_7134**	pBVIE02	1.76	5.28E-03	hypothetical protein	Function unknown
**Bcep1808_6430**	chr 3	1.75	1.26E-02	AsnC family transcriptional regulator	Transcription
**Bcep1808_6171** [k]	chr 3	1.74	1.33E-03	ABC transporter related	Defence mechanisms
**Bcep1808_7236** [r]	pBVIE02	1.73	4.61E-05	TniB family protein	Function unknown
**Bcep1808_7241**	pBVIE02	1.72	1.47E-03	cupin 2 domain-containing protein	Function unknown
**Bcep1808_2666**	chr 1	1.72	1.37E-18	phage integrase family protein	Replication, recombination and repair
**Bcep1808_3913**	chr 2	1.72	4.71E-04	ArsR family transcriptional regulator	Transcription
**Bcep1808_7034**	pBVIE02	1.71	6.84E-05	transcriptional regulator	Transcription
**Bcep1808_6157** [i]	chr 3	1.71	1.31E-07	cytochrome c oxidase, subunit I	Energy production and conversion
**Bcep1808_6223**	chr 3	1.71	2.89E-13	hypothetical protein	Function unknown
**Bcep1808_2920**	chr 1	1.71	6.76E-23	RNA polymerase factor sigma-32	Transcription
**Bcep1808_7234** [r]	pBVIE02	1.71	1.14E-03	integrase catalytic subunit	Replication, recombination and repair
**Bcep1808_6839**	pBVIE01	1.70	1.68E-14	hypothetical protein	Function unknown
**Bcep1808_3058**	chr 1	1.70	2.20E-06	bile acid:sodium symporter	General function prediction only
**Bcep1808_7284**	pBVIE02	1.69	3.10E-04	hypothetical protein	Function unknown
**Bcep1808_7295**	pBVIE02	1.69	1.42E-03	tungstate/molybdate transport system permease protein	Posttranslational modification, protein turnover, chaperones

**Bcep1808_2778**	chr 1	1.69	3.57E-09	galactonate dehydratase	Cell wall/membrane/envelope biogenesis
**Bcep1808_5444**	chr 2	1.69	1.41E-03	hypothetical protein	Function unknown
**Bcep1808_4906**	chr 2	1.68	9.30E-06	sigma-54 dependent trancsriptional regulator	Signal transduction mechanisms
**Bcep1808_7254**	pBVIE02	1.68	4.97E-03	hypothetical protein	Function unknown
**Bcep1808_4711**	chr 2	1.68	3.86E-02	hypothetical protein	Function unknown
**Bcep1808_0170** [a]	chr 1	1.68	3.85E-28	UBA/THIF-type NAD/FAD binding protein	Coenzyme transport and metabolism
**Bcep1808_0520**	chr 1	1.67	5.13E-24	phenylacetic acid degradation protein paaN	Energy production and conversion
**Bcep1808_0445**	chr 1	1.67	2.71E-37	hypothetical protein	Function unknown
**Bcep1808_2722** [e]	chr 1	1.66	6.59E-55	hydrophobe/amphiphile efflux-1 (HAE1) family protein	Defence mechanisms
**Bcep1808_2498**	chr 1	1.66	2.04E-17	outer membrane protein (porin)-like protein	Cell wall/membrane/envelope biogenesis
**Bcep1808_7265**	pBVIE02	1.66	4.71E-03	hypothetical protein	Function unknown
**Bcep1808_5492**	chr 3	1.66	2.24E-09	hypothetical protein	Function unknown
**Bcep1808_7270**	pBVIE02	1.64	5.66E-04	heavy metal translocating P-type ATPase	Inorganic ion transport and metabolism
**Bcep1808_7240**	pBVIE02	1.64	1.44E-03	hypothetical protein	Function unknown
**Bcep1808_4515** [f]	chr 2	1.64	2.43E-06	hypothetical protein	Function unknown
**Bcep1808_6331**	chr 3	1.64	1.47E-03	hypothetical protein	Function unknown
**Bcep1808_7210**	pBVIE02	1.64	9.21E-06	LysR family transcriptional regulator	Transcription
**Bcep1808_6186**	chr 3	1.64	6.12E-03	hypothetical protein	Function unknown

**Bcep1808_4514** [f]	chr 2	1.64	1.82E-10	hypothetical protein	Transcription
**Bcep1808_6856**	pBVIE01	1.63	5.70E-16	superfamily I DNA/RNA helicase-like protein	Replication, recombination and repair
**Bcep1808_6855**	pBVIE01	1.63	1.23E-07	hypothetical protein	Function unknown
**Bcep1808_7226**	pBVIE02	1.62	1.02E-02	hypothetical protein	Function unknown
**Bcep1808_7129**	pBVIE02	1.62	2.40E-02	DNA repair exonuclease-like protein	Replication, recombination and repair
**Bcep1808_5481**	chr 3	1.62	4.32E-24	cobyrinic acid a,c-diamide synthase	Cell cycle control, cell division, chromosome partitioning
**Bcep1808_2703**	chr 1	1.61	3.09E-04	xylulokinase	Carbohydrate transport and metabolism
**Bcep1808_6829**	pBVIE01	1.61	1.77E-07	hypothetical protein	Function unknown
**Bcep1808_7693**	pBVIE05	1.61	1.34E-07	hypothetical protein	Function unknown
**Bcep1808_7036**	pBVIE02	1.61	1.41E-03	integrase catalytic subunit	Replication, recombination and repair
**Bcep1808_2721** [e]	chr 1	1.61	4.81E-19	RND efflux system outer membrane lipoprotein	Cell wall/membrane/envelope biogenesis
**Bcep1808_6187** [n]	chr 3	1.61	1.32E-04	hemerythrin HHE cation binding domain-containing protein	Function unknown
**Bcep1808_5375**	chr 2	1.61	2.38E-07	glutamine synthetase	Amino acid transport and metabolism
**Bcep1808_7186**	pBVIE02	1.61	5.20E-02	heavy metal transport/detoxification protein	Inorganic ion transport and metabolism
**Bcep1808_7181**	pBVIE02	1.60	3.59E-02	hypothetical protein	Function unknown
**Bcep1808_7250**	pBVIE02	1.60	2.59E-03	hypothetical protein	Function unknown
**Bcep1808_7275**	pBVIE02	1.60	1.85E-02	hypothetical protein	Function unknown

**Bcep1808_6070**	chr 3	1.60	5.49E-06	hypothetical protein	Function unknown
**Bcep1808_6119** [g]	chr 3	1.59	2.28E-16	GCN5-related N-acetyltransferase	General function prediction only
**Bcep1808_6158** [i]	chr 3	1.59	5.75E-03	cytochrome c, class I	Energy production and conversion
**Bcep1808_7280**	pBVIE02	1.59	3.72E-02	hypothetical protein	Function unknown
**Bcep1808_6311**	chr 3	1.59	1.08E-03	FtsH-2 peptidase	Posttranslational modification, protein turnover, chaperones
**Bcep1808_0444**	chr 1	1.58	2.41E-13	resolvase domain-containing protein	Replication, recombination and repair
**Bcep1808_2828**	chr 1	1.58	5.03E-06	TetR family transcriptional regulator	Transcription
**Bcep1808_7249**	pBVIE02	1.58	7.49E-04	hypothetical protein	Function unknown
**Bcep1808_6320**	chr 3	1.57	2.37E-03	hypothetical protein	Defence mechanisms
**Bcep1808_7115**	pBVIE02	1.57	2.10E-02	hypothetical protein	Intracellular trafficking, secretion, and vesicular transport
**Bcep1808_6205**	chr 3	1.56	8.16E-06	histone family protein nucleoid-structuring protein H-NS	General function prediction only
**Bcep1808_1549**	chr 1	1.56	1.58E-04	UspA domain-containing protein	Signal transduction mechanisms
**Bcep1808_3305**	chr 1	1.56	3.36E-02	hypothetical protein	Function unknown
**Bcep1808_0751**	chr 1	1.56	6.06E-02	glutathione-dependent formaldehyde-activating protein	Function unknown
**Bcep1808_4884**	chr 2	1.55	3.99E-02	hypothetical protein	Function unknown
**Bcep1808_6723**	pBVIE01	1.55	2.20E-04	hypothetical protein	Function unknown
**Bcep1808_0750**	chr 1	1.55	8.45E-03	IclR family transcriptional regulator	Transcription
**Bcep1808_7272**	pBVIE02	1.55	8.12E-04	PHB de-polymerase domain-containing protein	Lipid transport and metabolism

**Bcep1808_4926**	chr 2	1.54	7.52E-06	chromosome replication initiation inhibitor protein	Transcription
**Bcep1808_3156**	chr 1	1.53	9.28E-03	aldehyde dehydrogenase	Energy production and conversion
**Bcep1808_2723** [e]	chr 1	1.52	8.51E-36	RND family efflux transporter MFP subunit	Cell wall/membrane/envelope biogenesis
**Bcep1808_7229**	pBVIE02	1.52	8.48E-02	hypothetical protein	Function unknown
**Bcep1808_3967**	chr 2	1.51	1.04E-02	TetR family transcriptional regulator	Transcription
**Bcep1808_7206**	pBVIE02	1.51	8.45E-03	transposase Tn3 family protein	Replication, recombination and repair
**Bcep1808_5371**	chr 2	1.50	7.85E-08	putative nicotinate phosphoribosyltransferase	Coenzyme transport and metabolism
**Bcep1808_6172** [k]	chr 3	1.50	9.31E-04	ABC-2 type transporter	Defence mechanisms
**Bcep1808_6761**	pBVIE01	1.50	3.46E-07	OmpA/MotB domain-containing protein	Cell wall/membrane/envelope biogenesis
**Bcep1808_2163**	chr 1	1.50	8.97E-05	hypothetical protein	Function unknown
**Bcep1808_7089**	pBVIE02	1.50	5.07E-03	hypothetical protein	Function unknown
**Bcep1808_7247**	pBVIE02	1.50	6.67E-03	hypothetical protein	Function unknown

Footnote: [1] Putative gene function annotation by Prokka (3); [2] COG, Cluster of orthologous groups category identified using the EggNOG database(4); [a - r] Differential expression of a gene operon.

46 **Supplemental Table S6**. Significantly down-regulated genes (≥ 1.5-fold change) of *B. vietnamiensis* strain G4 in response to sub-inhibitory (0.5
47 x MIC) of HEPB.

Gene ID	Replicon	log2 Fold Change	Adjusted p-value	Putative Gene function [1]	COG Category [2]
Bcep1808_6689 [h]	pBVIE01	-3.89	4.43E-35	hypothetical protein	Function unknown
Bcep1808_6686 [g]	pBVIE01	-3.63	8.33E-27	GntR family transcriptional regulator	Transcription
Bcep1808_6687 [g]	pBVIE01	-3.52	9.78E-27	electron transfer flavoprotein beta-subunit	Energy production and conversion
Bcep1808_2790	chr 1	-3.10	9.15E-12	glycerol kinase	Energy production and conversion
Bcep1808_4016	chr 2	-3.06	7.61E-06	GntR family transcriptional regulator	Transcription
Bcep1808_6688 [g]	pBVIE01	-3.01	1.24E-27	electron transfer flavoprotein, alpha subunit	Energy production and conversion
Bcep1808_6690 [h]	pBVIE01	-2.94	2.12E-17	amino acid permease-associated region	Amino acid transport and metabolism
Bcep1808_3523	chr 2	-2.85	6.09E-61	aldehyde dehydrogenase	Energy production and conversion
Bcep1808_6683	pBVIE01	-2.84	6.76E-23	extracellular ligand-binding receptor	Amino acid transport and metabolism
Bcep1808_2789	chr 1	-2.81	1.46E-06	glycerol-3-phosphate dehydrogenase	Energy production and conversion
Bcep1808_4978	chr 2	-2.70	3.38E-05	hypothetical protein	Function unknown

Bcep1808_1488 [d]	chr 1	-2.64	2.83E-05	response regulator receiver protein	Intracellular trafficking, secretion, and vesicular transport
Bcep1808_1486	chr 1	-2.56	4.82E-05	SAF domain-containing protein	Intracellular trafficking, secretion, and vesicular transport
Bcep1808_6679 [f]	pBVIE01	-2.47	1.85E-27	hypothetical protein	Function unknown
Bcep1808_4973	chr 2	-2.36	9.78E-16	putative zinc-containing dehydrogenase	Amino acid transport and metabolism
Bcep1808_6691	pBVIE01	-2.35	4.61E-05	extracellular ligand-binding receptor	Amino acid transport and metabolism
Bcep1808_4974	chr 2	-2.28	1.45E-05	porin	Cell wall/membrane/envelope biogenesis
Bcep1808_1485 [c]	chr 1	-2.12	5.73E-03	TadE family protein	Intracellular trafficking, secretion, and vesicular transport
Bcep1808_3473	chr 2	-2.12	2.38E-05	membrane protein-like protein	Function unknown
Bcep1808_1311	chr 1	-2.11	2.23E-03	hypothetical protein	Function unknown
Bcep1808_4025	chr 2	-2.08	1.77E-07	porin	Cell wall/membrane/envelope biogenesis
Bcep1808_1487 [d]	chr 1	-2.08	5.51E-05	type II and III secretion system protein	Intracellular trafficking, secretion, and vesicular transport
Bcep1808_5488	chr 3	-2.07	1.04E-09	adenylosuccinate synthetase	Nucleotide transport and metabolism
Bcep1808_2859	chr 1	-2.06	6.12E-09	L-lactate transport	Energy production and conversion

Bcep1808_6680 [f]	pBVIE01	-1.99	7.07E-26	FAD linked oxidase domain-containing protein	Energy production and conversion
Bcep1808_1489 [d]	chr 1	-1.98	6.15E-09	type II secretion system protein E	Intracellular trafficking, secretion, and vesicular transport
Bcep1808_5954 [e]	chr 3	-1.96	3.09E-04	phospholipid/glycerol acyltransferase	Lipid transport and metabolism
Bcep1808_2791	chr 1	-1.95	6.36E-03	MIP family channel protein	Carbohydrate transport and metabolism
Bcep1808_6682	pBVIE01	-1.94	6.65E-03	hypothetical protein	Function unknown
Bcep1808_0786	chr 1	-1.93	1.78E-03	hypothetical protein	Function unknown
Bcep1808_1484 [c]	chr 1	-1.91	8.34E-04	peptidase A24A, prepilin type IV	Posttranslational modification, protein turnover, chaperones
Bcep1808_1668	chr 1	-1.88	1.98E-36	saccharopine dehydrogenase	Amino acid transport and metabolism
Bcep1808_5326	chr 2	-1.88	1.08E-05	response regulator receiver protein	Signal transduction mechanisms
Bcep1808_1482 [b]	chr 1	-1.85	1.60E-03	hypothetical protein	Function unknown
Bcep1808_4091	chr 2	-1.84	3.44E-08	triple helix repeat-containing collagen	Function unknown
Bcep1808_5964 [e]	chr 3	-1.83	7.34E-03	3-oxoacyl-ACP synthase	Lipid transport and metabolism
Bcep1808_1303	chr 1	-1.82	2.86E-03	hypothetical protein	General function prediction only
Bcep1808_1300 [a]	chr 1	-1.80	8.51E-03	hypothetical protein	Function unknown
Bcep1808_5173	chr 2	-1.79	3.32E-05	methyl-accepting chemotaxis sensory transducer	Cell motility

Bcep1808_2793	chr 1	-1.78	1.22E-05	3,4-dihydroxy-2-butanone 4-phosphate synthase	Coenzyme transport and metabolism
Bcep1808_1490 [d]	chr 1	-1.78	1.11E-03	type II secretion system protein	Intracellular trafficking, secretion, and vesicular transport
Bcep1808_1480	chr 1	-1.77	5.34E-04	polypeptide-transport-associated domain-containing protein	Intracellular trafficking, secretion, and vesicular transport
Bcep1808_1070	chr 1	-1.76	3.45E-02	pirin domain-containing protein	General function prediction only
Bcep1808_3541	chr 2	-1.75	5.56E-04	EmrB/QacA family drug resistance transporter	Carbohydrate transport and metabolism
Bcep1808_5041	chr 2	-1.74	6.62E-04	virulence factor family protein	Intracellular trafficking, secretion, and vesicular transport
Bcep1808_3998	chr 2	-1.74	1.42E-04	hypothetical protein	Function unknown
Bcep1808_4006	chr 2	-1.72	4.19E-02	hypothetical protein	Function unknown
Bcep1808_4090	chr 2	-1.69	1.94E-03	hypothetical protein,Isoprenylcysteine carboxyl methyltransferase (ICMT) family	Function unknown
Bcep1808_2821	chr 1	-1.68	2.11E-05	cytochrome c oxidase subunit III	Energy production and conversion
Bcep1808_5963 [e]	chr 3	-1.68	3.50E-02	lipoprotein transmembrane	General function prediction only
Bcep1808_1491 [d]	chr 1	-1.67	1.08E-05	type II secretion system protein	Cell motility
Bcep1808_5960 [e]	chr 3	-1.66	5.38E-04	histidine ammonia-lyase	Amino acid transport and metabolism
Bcep1808_3235	chr 1	-1.64	4.01E-02	hypothetical protein	Function unknown

Bcep1808_6367	chr 3	-1.64	1.28E-02	YadA domain-containing protein	Intracellular trafficking, secretion, and vesicular transport
Bcep1808_3087	chr 1	-1.63	5.78E-02	FAD dependent oxidoreductase	Amino acid transport and metabolism
Bcep1808_5026	chr 2	-1.63	5.78E-02	major facilitator transporter	Carbohydrate transport and metabolism
Bcep1808_1481 [b]	chr 1	-1.62	1.27E-02	hypothetical protein	Function unknown
Bcep1808_4001	chr 2	-1.62	1.37E-02	acyl-homoserine lactone synthase (*bviI*)	Signal transduction mechanisms
Bcep1808_4771	chr 2	-1.61	2.10E-02	hypothetical protein	Function unknown
Bcep1808_1296 [a]	chr 1	-1.59	1.29E-03	bacteriophage Mu tail sheath family protein	General function prediction only
Bcep1808_7426	pBVIE03	-1.59	4.65E-02	hypothetical protein	Function unknown
Bcep1808_5959 [e]	chr 3	-1.58	3.58E-03	glycosyl transferase family protein	General function prediction only
Bcep1808_1299 [a]	chr 1	-1.58	5.46E-02	hypothetical protein	Function unknown
Bcep1808_3509	chr 2	-1.57	7.04E-02	silent information regulator protein Sir2	Transcription
Bcep1808_3360	chr 2	-1.55	4.75E-02	integral membrane sensor hybrid histidine kinase	Signal transduction mechanisms
Bcep1808_3370	chr 2	-1.52	2.62E-03	leucine/isoleucine/valine transporter ATP-binding subunit (*livG)*	Amino acid transport and metabolism

Bcep1808_2198	chr 1	-1.52	1.80E-02	17 kDa surface antigen	Cell wall/membrane/envelope biogenesis
Bcep1808_1483	chr 1	-1.51	1.34E-07	Flp/Fap pilin component	Intracellular trafficking, secretion, and vesicular transport
Bcep1808_4185	chr 2	-1.50	8.30E-02	AraC family transcriptional regulator	Transcription
Bcep1808_3818	chr 2	-1.50	8.06E-03	hypothetical protein	Function unknown

Footnote: [1] Putative gene function annotation by Prokka (3); [2] COG, Cluster of orthologous groups category identified using the EggNOG database (4); [a - h] Differential expression of a gene operon.

Supplemental Table S7. PCR primers used in this study

Primer	Primer sequence 5' to 3'	Reference
Primer 1	TTTTTACACTGATGAATGTTCCG	Jones *et al.* (5)
Primer 2b	GGC CAC GCG TCG ACT AGT ACN NNN NNN NNN ACG CC	Manoil *et al.* (6)
Primer 3	CGG ATTACAGCCGGATCCCCG	Jones *et al.* (5)
Primer 4	GGC CAC GCG TCG ACT AGT AC	Manoil *et al.* (6)

Supplemental References

1. EU. 2009. Regulation (EC) No 1223/2009 of the European Parliament and of the Council of 30 November 2009 on cosmetic products. OJ L 342 22.12.2009. http://data.europa.eu/eli/reg/2009/1223/2019-11-27.
2. Rushton L, Sass A, Baldwin A, Dowson CG, Donoghue D, Mahenthiralingam E. 2013. Key role for efflux in the preservative susceptibility and adaptive resistance of Burkholderia cepacia complex bacteria. Antimicrobial Agents and Chemotherapy 57:2972-2980.
3. Seemann T. 2014. Prokka: Rapid prokaryotic genome annotation. Bioinformatics 30:2068-2069.
4. Jensen LJ, Julien P, Kuhn M, von Mering C, Muller J, Doerks T, Bork P. 2008. eggNOG: Automated construction and annotation of orthologous groups of genes. Nucleic Acids Research 36:D250-D254.
5. Jones BV, Young R, Mahenthiralingam E, Stickler DJ. 2004. Ultrastructure of Proteus mirabilis swarmer cell rafts and role of swarming in catheter-associated urinary tract infection. Infection and Immunity 72:3941-3950.
6. Manoil C. 2000. Tagging exported proteins using Escherichia coli alkaline phosphatase gene fusions, vol 326, p 35-47.
